# Can dry rivers provide a good quality of life? Integrating beneficial and detrimental nature’s contributions to people over time

**DOI:** 10.1007/s13280-024-02072-x

**Published:** 2024-09-24

**Authors:** Néstor Nicolás-Ruiz, María Luisa Suárez, María Rosario Vidal-Abarca, Cristina Quintas-Soriano

**Affiliations:** 1 Department of Ecology and Hydrology, Faculty of Biology, Campus of International Excellence ‘Campus mare Nostrum’, University of Murcia, Campus of Espinardo, 30100 Murcia, Spain; 2 Andalusian Centre for the Assessment and Monitoring of Global Change (CAESCG), Department of Biology and Geology, University of Almeria, 04120 Almería, Spain; 3 FRACTAL Collective, San Remigio 2, 28022 Madrid, Spain

**Keywords:** Ecosystem disservices, Ecosystem services, Ephemeral streams, Human well-being, Interviews, Social perception

## Abstract

Although dry rivers only flow after heavy rainfall, recent studies have evidenced that they provide benefits to people. However, part of society undervalues these benefits, raising the question of whether they contribute to a good quality of life (GQL). We hypothesised that the benefits of dry rivers contribute to GQL of local communities as long as these benefits are not altered by human disturbance. Local communities closely associated with three dry rivers in Murcia (Spain) were interviewed. The relationships between benefits and GQL, and between detriments and GQL were described. Different benefits and detriments have different impacts on GQL dimensions. Social perceptions of the contribution of benefits and detriments to GQL vary between communities and individuals, and change over time. This implies the development of social studies to ensure inclusive management. Incorporating detriment co-production into research can raise people’s awareness of the role that human activities play in maintaining GQL.

**Supplementary Information:**

The online version contains supplementary material available at 10.1007/s13280-024-02072-x.

## Introduction

More than a third of the world’s population lives in drylands where freshwater is scarce (Adeel et al. 2005). Drylands cover 45% of the Earth's land surface, predicted to increase as a result of climate change and land degradation (Prăvălie 2016). They are located in dry sub-humid, semi-arid, arid and hyper-arid climates. Here, freshwater ecosystems are essential for people’s livelihoods, with dry rivers being the most common (Messager et al. 2021). A dry river is defined as a channel with no surface water flow except after heavy rainfall or snowmelt, which often causes flash-floods. As these floods last a few hours or days, the exchange between surface and groundwater is brief, and the predominant life forms are terrestrial (Vidal-Abarca et al. 2020).

Despite the lack of water flow, dry rivers provide material (supporting people’s physical existence), non-material (supporting people’s psychological existence) and regulating (modulating the environmental conditions that enable human life) benefits to people (Díaz et al. 2015). For instance, material benefits include food (e.g. grains), materials (e.g. vegetable fibres) and herbal medicines (e.g. thyme); non-material benefits include experiences (e.g. hiking), knowledge (e.g. environmental education) and cultural identity (e.g. hydraulic heritage); and regulating benefits include freshwater (e.g. aquifer recharge), fertile soils (e.g. floodplains) and microclimates (e.g. soft temperatures) (Jacobson et al. 1995; Steward et al. 2012; Koundouri et al. 2017). Most of these benefits are co-produced between the natural and social systems of dry rivers (Comberti et al. 2015; Palomo et al. 2016). The natural system provides environmental conditions (e.g. soil fertility) that the social system uses to obtain benefits (e.g. crops), thanks to their local ecological knowledge acquired over generations (Vidal-Abarca et al. 2022). Nevertheless, social perception studies reveal that society fails to recognise these benefits or show a willingness to preserve them (García-Llorente et al. 2012; Koundouri et al. 2017; Leigh et al. 2019; Rodríguez-Lozano et al. 2020). This raises the question of whether the benefits of dry rivers really contribute to a good quality of life.

A Good Quality of Life (GQL) can be defined as a long and fulfilled human life, free from poverty and disease, and with access to freedoms and rights (Díaz et al. 2015). A fulfilled human life involves to meet physical human needs such as food, water and livelihood security (i.e. material GQL dimensions) and experiential human needs such as good social relationships, equity and freedom to exercise spirituality (i.e. non-material GQL dimensions) (Rogers et al. 2012; Brondízio et al. 2019). The GQL dimensions prioritised to achieve a fulfilled life depend on the value system of each individual, society and culture (Shin et al. 2019) and consequently vary across spatial (continents, countries, regions) and temporal scales (centuries, decades, years) (Pascual et al. 2017; Liu et al. 2023). A value system can be defined as a set of principles, judgments and opinions that determine the nature-human relationships and their importance to humans. These systems are developed from knowledge, experience, and connection with nature (Anderson et al. 2022).

The fact that GQL is a context-dependent state may explain why some studies praise dry river benefits, while others underestimate them. Most reviews of dry river benefits collect evidence from local communities (e.g. Koundouri et al. 2017; Vidal-Abarca et al. 2022). These are small communities whose livelihood, identity, knowledge and values are closely linked to nature (Brondízio et al. 2019). In contrast, most social perception studies correspond with non-strictly local communities (e.g. Rodríguez-Lozano et al. 2020). To illustrate, for the local Turkana community in Kenya, nomadic pastoralism integrates economic, social and spiritual values associated with both material and non-material GQL dimensions. In contrast, for mainstream Kenyan society, it has mainly a small economic value associated with material GQL dimensions (Teria Ng’asike 2010; Betti 2018). Thus, it can be hypothesised that dry river benefits are perceived and contribute more to the quality of life of local communities than those whose values and knowledge are less associated with nature (Anderson et al. 2022).

Most case studies assume nature’s benefits contribute to a GQL without first testing or being part of a hypothesis (Cruz-Garcia et al. 2017). These studies fail to recognise that relationships between benefits and GQL change over time and occur at different spatial scales. Failure to discuss the social, cultural and economic context can lead to three errors in the interpretation of results. First, all social communities perceive benefits and GQL in the same way. Second, potential benefits of dry rivers always contribute in the same way to different GQL dimensions (McMichael et al. 2005; Daw et al. 2011). And third, GQL management measures are extrapolated to any dry river.

Another topic that is often overlooked in studies of dry rivers, and in all ecosystems in general (Shackleton et al. 2016; Blanco et al. 2019), is that nature provides not only benefits but also detriments that negatively affect to a GQL (Pastor et al. 2022). The only commonly reported detriment of dry rivers is the material and human damage caused by flash-floods (Quiñonero-Rubio et al. 2011; Diakakis 2022). The set of detriments and benefits from nature can be grouped under the term ‘Nature’s Contributions to People’ (NCP), as proposed by Díaz et al. (2015) and applied throughout the manuscript. Human factors that affect the NCP provision are known as drivers of change (MEA 2005; Rounsevell et al. 2010; Balvanera et al. 2019). Two distinct types of drivers of change can be identified: direct (acting directly on nature and its benefits, such as land-use change) and indirect (acting by leveraging direct drivers, such as institutional factors) (Chiu et al. 2017; Vidal-Abarca et al. 2022). These drivers can reduce beneficial NCP and increase detrimental NCP, affecting the perception of how dry rivers contribute to a GQL. Thus, a second hypothesis that may explain the social undervaluation of dry rivers is that their potential benefits could be altered and unmasked by the detrimental effects caused by drivers of change.

This study forms part of a larger research project that aims to gain a deeper understanding of the complex relationships between dry rivers and their associated social communities. These systems are defined as social-ecological systems (Biggs et al. 2021). While a preliminary study assessed the capacity of dry rivers to provide benefits to people (Nicolás-Ruiz et al. 2023), this second study aims to analyse the capacity of dry rivers to provide a GQL. To this end, four specific objectives were addressed: (i) to identify detriments of dry rivers, (ii) to characterise how the social system of dry rivers perceives a GQL, (iii) to determine the relationships between benefits and detriments of dry rivers and GQL dimensions, (iv) to assess changes in social perceptions of GQL over time.

## Materials and methods

### Study area

**Fig. 1:**
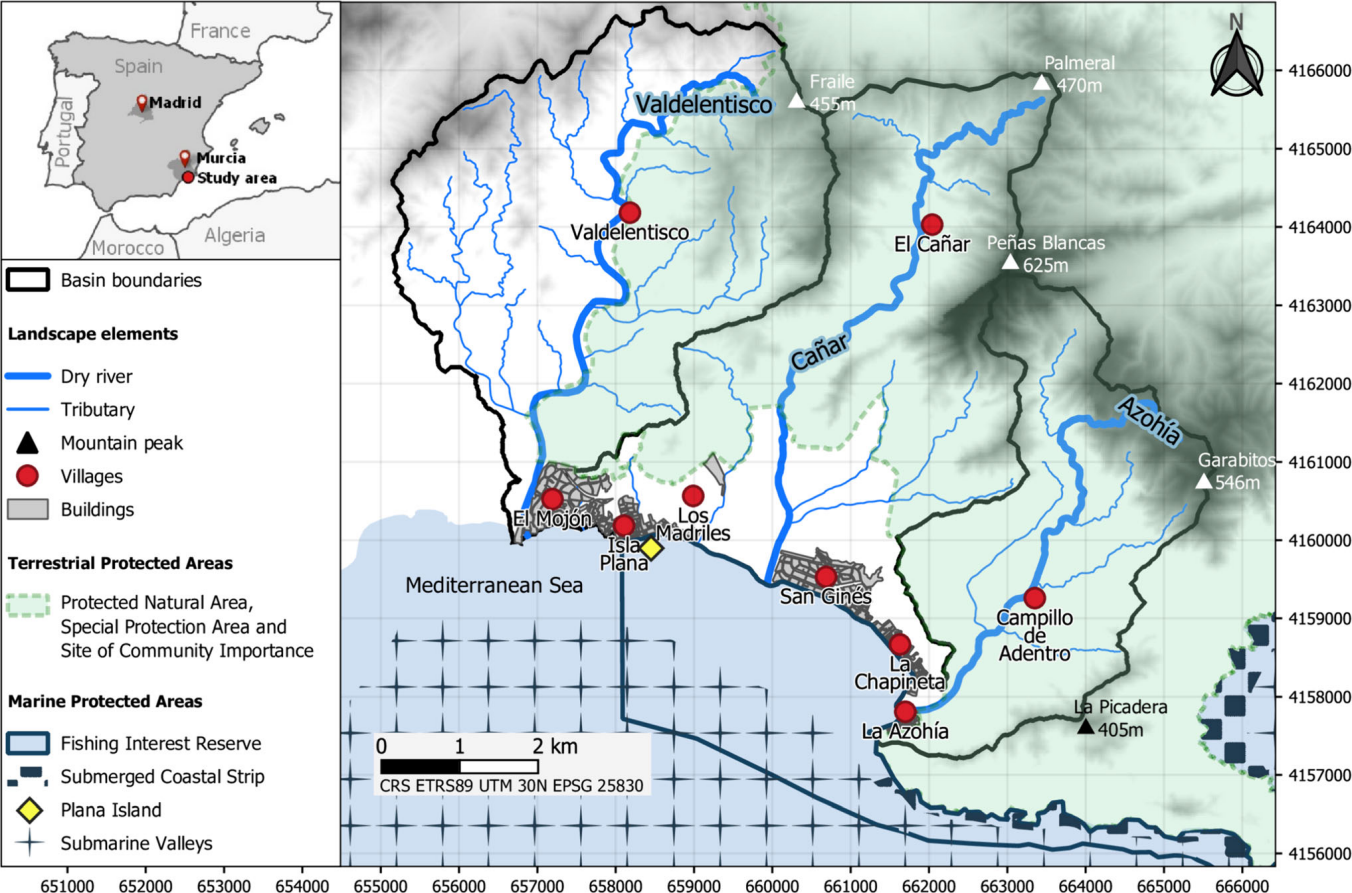
Location of the Region of Murcia in south-eastern Spain and map of the study area. The black line demarcates the study basins: Valdelentisco, El Cañar and La Azohía. The thick blue lines are the dry rivers and the thin blue lines are their tributaries. The triangles are the highest mountain peaks. The red circles are the villages. The grey rectangles are urban areas. Terrestrial and marine protected areas are represented by different symbols. CRS = Coordinate Reference System

The study area is located in the southeast of Spain in the Region of Murcia. It belongs to the municipalities of Cartagena and, to a lesser extent, Mazarrón. It comprises three small mountain basins that flow into the Mediterranean Sea: Valdelentisco (22.62 km^2^), El Cañar (22.96 km^2^) and La Azohía (13.29 km^2^). Each basin has a main dry river with the same name and several tributaries (Fig. 1). The climate is hot semi-arid with an average annual temperature of 17 °C and rainfall of 225 mm (DGMN 2006). Most of the study area is covered by Mediterranean shrub, esparto grasses, pine forests resulting from reforestation, and terraced rainfed crops with natural hedges (DGMN 2006).

Two socio-economic and geographical areas can be distinguished: the coastal strip in the lower part of the basins and the inland area in the upper part of the basins (Fig. 1). The coastal strip includes six urban areas where most people live (1577 inhabitants), while the inland area includes three small villages and scattered hamlets where a few families live (112 inhabitants; AdC 2021). In the inland area the main economic activities are traditional rainfed agriculture (e.g. carob and almond trees) and pastoralism (e.g. sheep, goats), while the main socio-cultural activities are hiking and pilgrimages. Other activities include beekeeping to supplement family income, the use of vegetable fibres as building materials (e.g. *Stipa tenacissima* L*., Chamaerops humilis* L.), the harvesting of aromatic plants for herbalism, floristry or food spices (e.g. *Thymus hyemalis* LANGE*, Rosmarinus officinalis* L.), and hunting for leisure and subsistence (e.g. partridges, rabbits). In the coastal strip the main socio-economic activities are beach tourism, greenhouse crops (e.g. tomatoes) and traditional fishing. The main environmental conflicts are the intensification of agriculture, the pollution of groundwater by agriculture and livestock, the dumping of rubbish by visitors, and the land abandonment due to low rentability and urban development (DGMN 2006; Nicolás-Ruiz et al. 2023).

The study area is characterised by the preservation of its natural habitats (e.g. Mediterranean scrub) and socio-cultural heritage (e.g. agricultural terraces). This is why we selected this area for the study. The majority of the study area is protected at the regional (Protected Natural Area) and European levels (Special Protection Area and Site of Community Importance) under the designation Cabo Tiñoso, La Muela and Roldán. The surrounding marine environment is also protected at national level (Marine Reserve of Fishing Interest) and at European level by the Natura 2000 Network (three Special Areas of Conservation, including the islands and islets of the Mediterranean Sea, the submerged coastal strip of the Region of Murcia, and the submarine valleys of Mazarrón) (Fig. 1).

### Sampling and interview design

We interviewed 37 representatives from 10 of the most influential social groups in the study area: neighbours (7), non-profit organisations (6), environmental managers (5), crop farmers (4), researchers (4), tourism sector (3), hunters (2), fishermen (2), livestock farmers (2) and neighbourhood councils (2) (Table S1). The social groups were determined according to the main socio-economic (e.g. agriculture, livestock) and socio-cultural (e.g. hiking, pilgrimages) activities of the social system in the dry rivers. These social groups were chosen to capture the widest range of interests and perceptions. They were identified prior to the interviews by reviewing local literature (e.g. DGMN 2006) and visiting the study area.

As part of the population resided or operated temporarily in the study basins, the inner hamlets were scattered, and a list of potential participants was not available, representatives of social groups were recruited through snowball sampling. This involved asking participants to introduce the researcher to others who met the inclusion criteria for the study (Kirchherr and Charles 2018; Knott et al. 2022). Participants were selected to encompass the widest possible range of genders, ages, occupations and educational backgrounds (Table S2) in order to minimise perceptual biases (see section: Limitations and methodological considerations). At least two representatives of each social group were interviewed. Group and sample sizes were determined based on the point of information saturation for detriments, GQL dimensions, and drivers of change (see section: Limitations and methodological considerations).

We designed a semi-structured interview around five topics: benefits, social conflicts, detriments, GQL, and drivers of change (Table S3). The first two topics were addressed in a preliminary study of the research (Nicolás-Ruiz et al. 2023), whose results on dry river benefits were applied in this manuscript (Table S4). The current study primarily addresses the third and fourth topics, with a lesser focus on the fifth topic. Each topic included a series of open-ended questions, which, unlike closed-ended questions, encourage more elaborate responses and contextual reflection (Knott et al. 2022). All open-ended questions were tested with participants identified during preliminary visits to the study area (e.g. goatherds, hikers). This interview methodology has been used successfully in numerous studies on nature’s benefits (Asah et al. 2014; Klain et al. 2014; Gould et al. 2015; Cheng et al. 2019; Topp et al. 2022).

The question linked to the first objective of the manuscript (dry river detriments) was as follows: Do dry rivers harm you in anyway? The questions linked to the second objective of the manuscript (GQL dimensions) were as follows: Are you happy here? How would you rate your quality of life? The questions linked to the third objective of the manuscript (relationships between benefits or detriments and GQL dimensions) were as follows: How do dry river benefits contribute your well-being? What aspects of your well-being do dry rivers fulfil? How do detriments of dry rivers affect your well-being? What aspects of your well-being are affected by these harms? The questions linked to the fourth objective of the manuscript (changes in GQL perception over time) were as follows: Have the benefits and detriments of dry rivers changed over last decades? What do you think are the reasons for these changes? How have these changes affected your well-being?

The interviews were conducted between December 2019 and March 2020. The snowball sampling started with the representatives of the neighbourhood councils and land managers, whose testimonies helped to identify the representatives of the other social groups. The first contact with the representatives was made by phone, email or on-site visit to inform them about the research, the importance of citizen participation and to ask for their cooperation in a face-to-face interview. The second contact with them was the interview itself at a pre-arranged time and place. All social group representatives agreed to be interviewed by means of a consent form, which informed them about the dynamics of the interview, data protection, audio recording, taking photographs and contact details. Their anonymity was protected at all stages of the research. The interviews were captured on a voice recorder and lasted on average 45 min.

### Interview data analysis

**Fig. 2:**
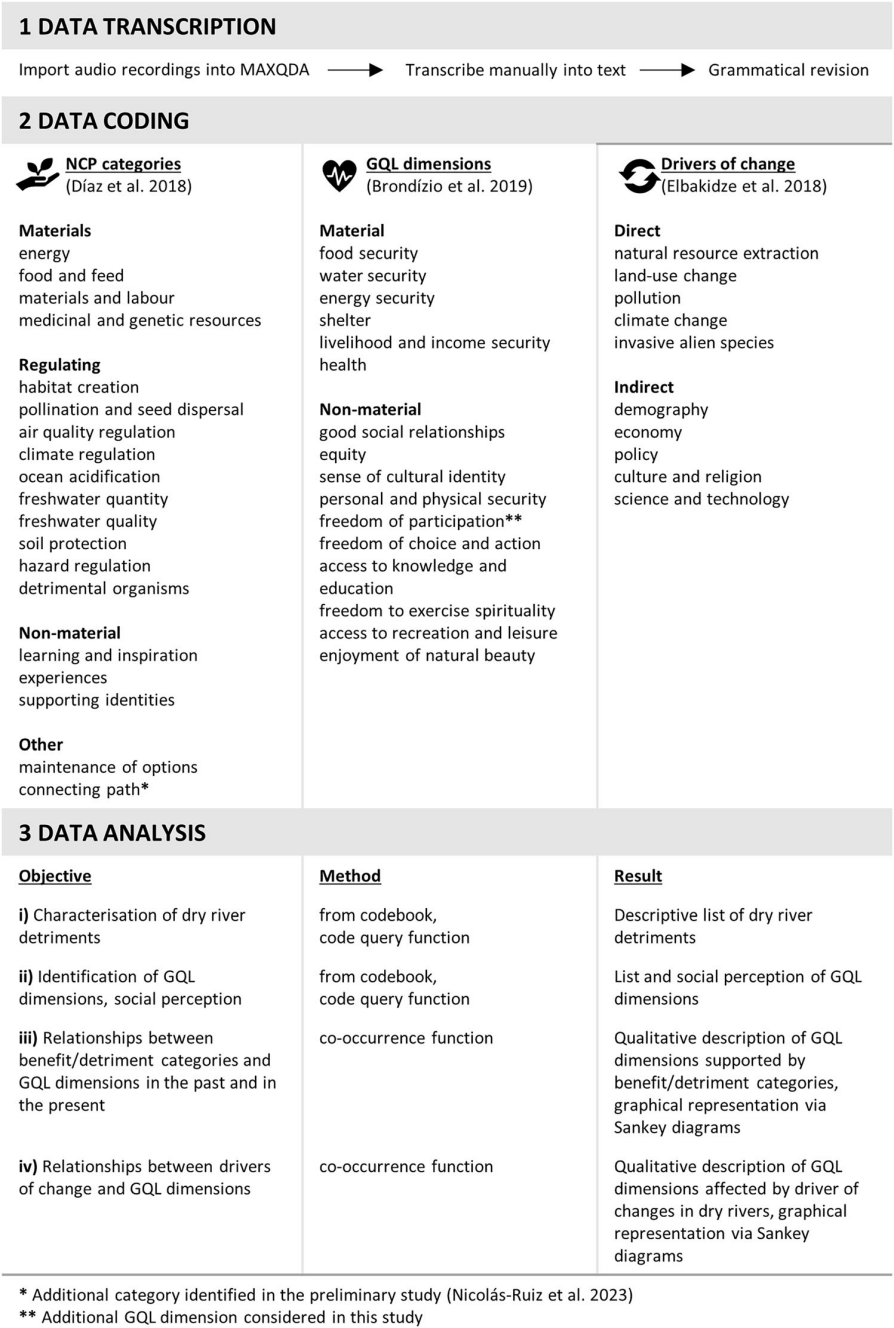
Method for analysing the interviews based on Knott et al. (2022): data transcription, data coding and data analysis. The categories used in the coding process are specified

The interview data were systematically analysed using the method proposed by Knott et al. (2022) for in-depth interviews in the social sciences. The method consists of three steps: data transcription, data coding and data analysis (Fig. 2). These steps were conducted using a software specialised in the analysis of qualitative data: MAXQDA Analytics Pro 2020 (Kuckartz and Rädiker 2019; Rädiker and Kuckartz 2020). Data transcription consisted of transcribing the interview audio recordings into text. The transcription focussed on the manifest content of the interviews without considering pauses, repetitions and false starts.

Data coding consisted of identifying and classifying transcript text segments of interest for the study objectives (i.e. sentences related to detriments, GQL dimensions, and drivers of change) by assigning them identification codes. The result is a codebook that registers and classifies the interviewees' quotes in different NCP, GQL and driver categories. The classification systems proposed by the Intergovernmental Science-Policy Platform on Biodiversity and Ecosystem Services (IPBES; Díaz et al. 2018) were employed to code these themes. This framework describes the relationship between natural and social systems through the benefits and detriments they provide, the GQL dimensions, and the drivers of change (Díaz et al. 2015). Its classification systems, unlike those proposed by other frameworks (e.g. MEA 2005; TEEB 2010; Potschin and Haines-Young 2011), have been constructed under different scientific disciplines (natural, social, engineering sciences), stakeholders (governments, civil society, international organisations) and knowledge systems (Western science, local knowledge, indigenous wisdom), making it more inclusive and representative (Díaz et al. 2015; IPBES 2018a, 2018b, 2018c, 2018d). Furthermore, given that the IPBES framework groups benefits and detriments under the NCP concept, they fall into the same categories and are therefore comparable.

The generalising perspective of the IPBES framework distinguishes 18 NCP categories grouped into three clusters: regulating, material and non-material NCP (Díaz et al. 2018; Table S4); 15 GQL dimensions grouped into two clusters: material and non-material dimensions (Brondízio et al. 2019; Table S5), and 10 driver types grouped in two clusters: direct and indirect drivers (Elbakidze et al. 2018; Table S6). An additional NCP category was created to code a benefit not included by the IPBES framework, but evidenced by several studies: the use of dry riverbeds for access to villages and fields (Gómez et al. 2005; Steward et al. 2012; Nicolás-Ruiz et al. 2023). An additional GQL category was also considered: freedom of participation. The IPBES framework includes this category within good social relationships (Brondízio et al. 2019). However, the two dimensions were coded separately in order to look more closely at the freedoms of the social system. Note that beneficial NCP categories, which are necessary for the analysis of relationships with GQL dimensions, were coded in the preliminary study (Nicolás-Ruiz et al. 2023; Table S4). Only text segments that described a cause-effect relationship between two topics (i.e. NCP categories, GQL dimensions, driver types) were coded with two codes.

Coded sentences with NCP categories were also categorised with two temporal codes: past (sentences expressed by interviewees in past) and present (sentences expressed by interviewees in present). To identify the GQL dimensions that are covered or not covered by dry rivers, two codes were created: positive (dimensions that interviewees perceive positively as being covered) and negative (dimensions that interviewees perceive negatively as not being covered).

Data analysis entailed the processing of the coded text segments in order to arrive at findings. To address the detrimental NCP (objective i) and the GQL dimensions (objective ii), the coded categories for each variable were listed from the codebook. Subsequently, these categories were qualitatively described and exemplified through verbatim quotes of interviewees. To conduct the description, the testimonies that reported the interviewees on each category were compiled using the code query function of MAXQDA, and then synthesised through a comprehensive lecture. Furthermore, the number of interviewees reporting each category was counted from the frequency table generated by the previous function.

A code co-occurrence function was used to determine the relationships, firstly, between beneficial NCP categories and GQL dimensions (objective iii), secondly, between detrimental NCP and GQL dimensions (objective iii) and, thirdly, between drivers of change and GQL dimensions (objective iv). The resulting relationships were plotted through flow diagrams using SankeyMATIC^1^.

In the two first cases, the co-occurrence function identified the coded text segments with a beneficial or detrimental NCP and a GQL dimension. From these text segments, the function developed a matrix in which each element represents a particular relationship between an NCP category and a GQL dimension. Each element was linked to interviewee statements that refer to it, as well as information on the number of interviewees who reported that particular relationship. Such testimonies were used to describe how material and non-material GQL dimensions were supported or affected by the beneficial and detrimental NCP, respectively. In both cases, this function was repeated twice to describe the different relationships between past NCP and GQL dimensions and between present NCP and GQL dimensions.

In the third case, the co-occurrence function identified the coded text segments with a driver of change and a GQL dimension. From these text segments, the function developed a matrix in which each element represents a particular relationship between a driver of change and a GQL dimension. Each element was linked to interviewee statements that refer to it, as well as information on the number of interviewees who reported that particular relationship. Such testimonies were used to describe the drivers of dry rivers and how they affect each material and non-material GQL dimension.

## Results

### Detrimental NCP

Four detrimental NCP categories were reported by interviewees: regulation of hazards and extreme events, regulation of detrimental organisms, regulation of freshwater quantity, location and timing, and physical and psychological experiences. Of these, the former was reported by the largest number of interviewees (Table 1). They claimed to feel vulnerable in the face of large flash-floods, droughts and fires, which jeopardised their livelihoods. The second detriment was related to wild animal attacks (e.g. wild boars, rabbits), pests (e.g. *Thaumetopoea pityocampa* Denis & Schiffermüller) and invasive alien species (e.g. *Arundo donax* L.), which cause damage to farming infrastructures, recreation areas and forest habitats. The third detriment was related to small flash-floods, which temporarily disrupted the connecting pathways between villages and agricultural fields. Finally, some interviewees reported unpleasant physical and psychological experiences due to the presence of dust and plants obstructing paths.

**Table 1:** Categories of detrimental nature’s contributions to people (NCP) identified in the study area. The number of interviewees who reported each category is provided in brackets (*n*). The categories are described using examples and verbatim quotes from the interviews. The material (M) and non-material (NM) GQL dimensions affected by each detriment are also indicated

Detrimental NCP categories	Examples	Verbatim quotes	Interviewees	GQL dimensions
Regulation of hazards and extreme events (*n* = 14)	Large flash-floods	“One morning there was a person who had to go to the hospital but couldn’t because the dry river was flowing and caused a 70 cm sinkhole in the road. This always occurs when it rains heavily”	8_ORG_S2	Personal and physical security (NM)
	Fires	“Uncut reeds [*Arundo donax* L.] are very bad because in years of drought they dry out and can catch fire and cause the whole El Cañar to burn down”	1_CRO_T3	Personal and physical security (NM)
	Droughts	“Drought is deadly. When I was a child there was a tremendous drought. My father had to give up farming and look for another job”	1_CRO_T2	Livelihood and income security (M)
Regulation of detrimental organisms (*n* = 14)	Pests	“Pines breed a worm called *procesionaria* [*Thaumetopoea pityocampa* Denis & Schiffermüller]. You touch it and it itches. I and two others went to the emergency room for three years”	1_CRO_T3	Health (M)
	Animal attacks	“Wild boar is the most damaging species in the three basins. Almost everybody hates it. It is an animal that does damage because of its size and behaviour. It damages the stone walls of the farm terraces and this has a negative impact on people”	8_ORG_E2	Personal and physical security (NM)
	Invasive alien species	“Many paths, farm terraces and traditional irrigation ditches have deteriorated because they have been invaded by aggressive vegetation such as reeds [*Arundo donax* L*.*]”	3_NEI_B1	Personal and physical security (NM)
Regulation of freshwater quantity, location and timing (*n* = 12)	Small flash-floods	“When it rains, the people of El Cañar are cut off from communication, which has happened in the past and still happens today”	3_NEI_B2	Personal and physical security (NM)
Physical and psychological experiences (*n* = 5)	Unpleasant experiences	“In the spring it's great to see the *albaidas* [*Anthyllis cystoides* L.], but they’re not very well kept and you can't walk well at all. I would give up my land to make paths so that people can enjoy the walk”	1_CRO_T3	Access to recreation and leisure (NM)
	Unpleasant experiences	“People perceive dry rivers as uncomfortable because when it rains, they wash away everything and because they are full of ugly dusty weeds”	1_CRO_I1	Enjoyment of natural beauty (NM)

*ORG_S* non-profit social organisation, *CRO_T* traditional crop farmer, *ORG_E* non-profit environmental organisation, *NEI_B* neighbour born in the study basin, *CRO_I* industrial crop farmer

### GQL social perception

**Fig. 3:**
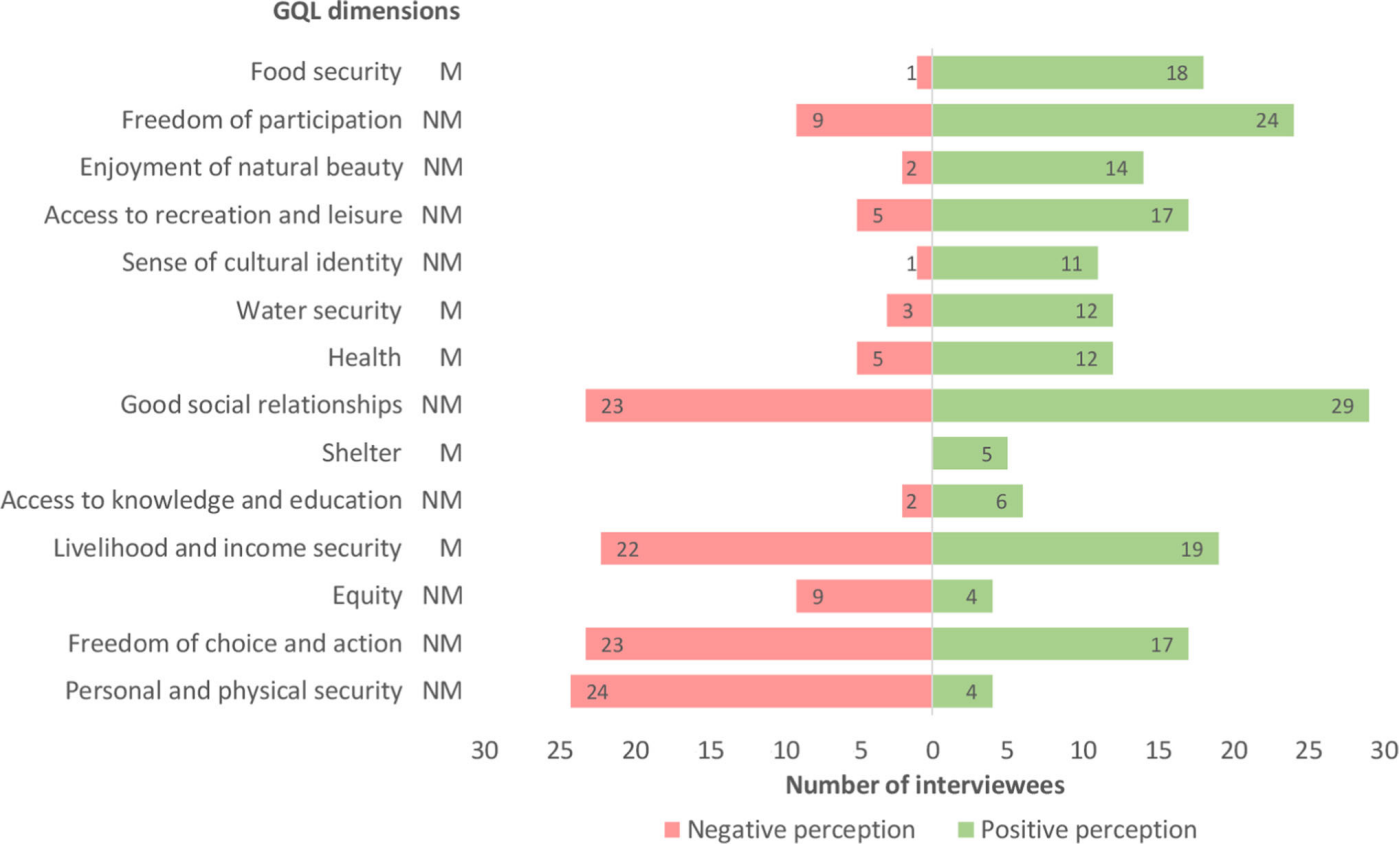
GQL dimensions as reported by interviewees. The red bars on the left indicate the number of interviewees who perceived that the GQL dimensions were not well covered by dry rivers (negative perception) and the green bars on the right indicate the number of interviewees who perceived that they were well covered by dry rivers (positive perception). GQL dimensions are ranked from best (top) to worst (bottom) perceived by interviewees based on the overall balance of positive and negative perceptions. M: GQL material dimension, NM: GQL non-material dimension

Interviewees referred to fourteen of the sixteen GQL dimensions studied: five material dimensions and nine non-material dimensions (Table 2). Energy security and freedom to exercise spirituality were not reported by any interviewee. Three social perception patterns were identified: dimensions mainly perceived positively (e.g. food security, freedom of participation), dimensions mainly perceived negatively (e.g. personal and physical security, freedom of choice and action), and dimensions perceived equally positively and negatively (e.g. good social relationships, livelihood and income security) (Fig. 3).

**Table 2:** Non-material GQL dimensions promoted by beneficial NCP, and their reporting frequency in terms of number of interviewees (*n*). GQL dimensions are exemplified by verbatim quotes from interviewees

GQL dimensions	*n*	Verbatim quotes	Interviewees
*Material GQL dimensions*			
Livelihood and income security	19	"There is a goatherd and his son who make their own cheese with denomination of origin, that is to say, there are few resources but they give you enough to live"	3_NEI_R2
		"Another livelihood has always been the esparto grass from which the women made *filete*^1^*"*	1_CRO_T3
Food security	18	"Here people will always be able to survive as long as they keep two square metres of land to grow two potatoes, two beans and an aubergine"	6_TOU_1
		"Dates have been an essential food for the survival of many people. Dry river palms were a way of feeding people who lived in isolation"	5_RES_3
Health	12	"My son comes at weekends and helps me prune the vines. He says it clears his mind and he likes it"	1_CRO_T2
		"A more contemporary function is to generate well-being through the enjoyment of landscapes and to generate mental and physical health"	2_MAN_L2
Water security	12	"In the old days, the dry rivers had water intakes for irrigation and they were very important because if you irrigated once, you were saved for several months"	6_TOU_2
		"It can go seven months without a drop of rain there, but you can survive on water from the *aljibes*^2^*.* You just have to count how many you need"	3_NEI_R2
Shelter	5	"The climate in the surrounding area is drier, but along dry rivers the climate is milder, allowing for human settlements"	2_MAN_L1
		"The dry riverbeds were a distribution of routes for goods. It was smuggling and everyone benefited from it because the orography made it possible to hide and the security forces got lost"	3_NEI_A1
*Non-material GQL dimensions*			
Good social relationships	29	“Women held *candangas*^3^, nightly gatherings to talk and keep each other company while they plaited esparto grass that they would later sell”	1_CRO_T3
		“The fact that traditions such as pilgrimages or festivals are preserved is both a cause and a consequence of a good relationship between all the people”	3_NEI_B1
Freedom of participation	24	“Cañar dry river belongs to two different neighbourhood councils, and although we are ideologically different, we are participating together in a working group to approve the new signposts and viewpoints towards the mountain and the dry river”	10_COU1
		“The neighbourhood associations in the area function quite well, they connect, they work together to convey the needs and feelings of all”	3_NEI_B2
Freedom of choice and action	17	“For the last eight years we have had more influence on decision-making because people are more committed to their identity and have seen that citizen participation is useful”	3_NEI_B1
		“Now the neighbours are working together with the administration to put up information panels and to hold nature days, and the results are being good”	3_NEI_B2
Access to recreation and leisure	17	“This area is extraordinary for small game because there aren't pines and the native feral partridge is fantastic”	4_HUN2
		“I like to walk along the dry river because you see lavender, rosemary, thyme, the smell they give off. If you leave the dry river all that doesn’t exist”	8_ORG_S2
Enjoyment of natural beauty	14	“I love this place. It has unrecognised values. For example, it has silence. There are very few landscapes with silence”	6_TOU_2
		“It's a pleasure to watch some of the predators. When I see the owls and eagles, I enjoy watching them”	4_HUN_2
Sense of cultural identity	11	“Even if I left here, the cattle and the land of my grandparents would be in my blood”	1_CRO_T3
		“For me the rambla is my childhood, fun and joy because I played there a lot. We always got our feet wet in the puddles”	10_COU1
Access to knowledge and education	6	“I've collected all kinds of plants. I learned to pick carobs and peas without breaking them thanks to my grandmother who taught me”	10_COU1
		“There is only one primary school here with fifty children and it does a good job because it introduces children to nature”	3_NEI_A1
Personal and physical security	4	“Places that don't have dry rivers flood, but here there have never been floods. Dry rivers drain rainwater and regulate flooding”	6_TOU_1
		“It is a very safe area to live in. We leave the yard door open at night and the grain on the threshing floor”	1_CRO_T2
Equity	4	“I admire the ability of the older people to organise and distribute resources among all the inhabitants”	3_NEI_B3
		“Dry rivers belonged to everyone, the herders with their livestock passed by, if there was a fig tree it belonged to everyone, it was the only public property”	6_TOU2

*RES* researcher, *HUN* hunter, *CRO_T* traditional crop farmer, *NEI_B* neighbour born in the study basin, *COU* neighbourhood council, *TOU* tourism sector, *NEI_A* foreign-born neighbour, *NEI_R* neighbour born in the Region of Murcia, *ORG_S* non-profit social organisation, *FIS* fisherman, *MAN_L* land environmental manager,

^1^Braided esparto rope with two strands

^2^Traditional water storage cisterns

^3^Traditional gatherings of women who weave esparto grass

### Relationships between beneficial NCP and GQL dimensions

**Fig. 4:**
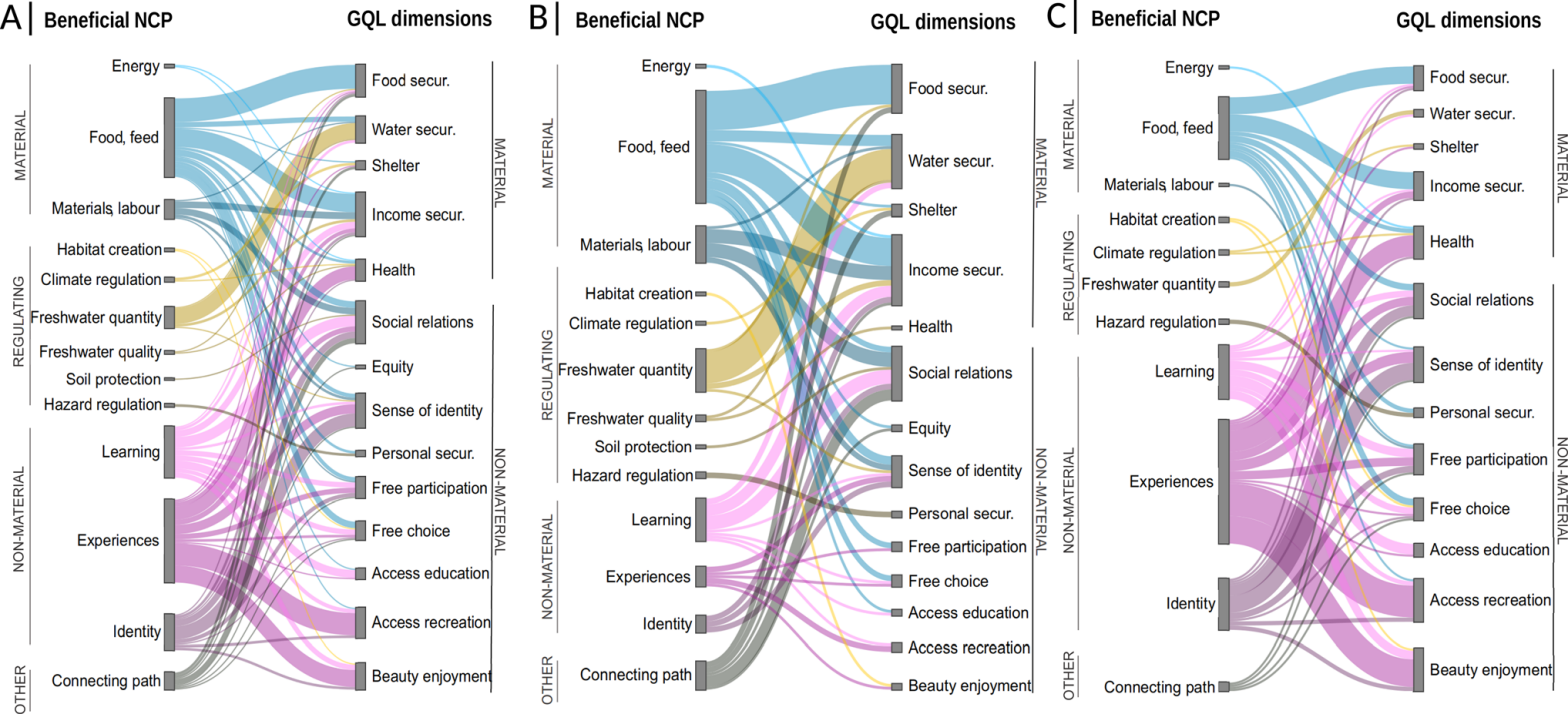
Relationships between beneficial NCP and GQL dimensions in the past and present (**a**), in the past only (**b**), and in the present only (**c**), as perceived by interviewees. The thickness of the lines indicates the number of interviewees who reported a relationship between a particular beneficial NCP and GQL dimension. The colours indicate the main NCP groups: material (blue), regulating (yellow), non-material (pink) and other (grey)

From the code co-occurrence function, seventy different relationships were identified between beneficial NCP and GQL dimensions, especially between non-material NCP and non-material dimensions (Fig. 4a). The relationships reported by the largest number of interviewees were food and feed with food security (*n* = 18), physical and psychological experiences with access to recreation and leisure (*n* = 17), and food and feed with livelihood and income security (*n* = 14) (Fig. S1).

#### Beneficial NCP and GQL material dimensions

Five GQL material dimensions promoted by beneficial NCP were identified through the code co-occurrence function: livelihood and income security, food security, water security, health, and shelter (Table 2, Fig. 4a). Access to food and forest resources provides a livelihood and income security for local people. They sell traditional products from agriculture (e.g. almonds), livestock (e.g. lambs), fish (e.g. sargo) and forestry (e.g. esparto grass). Neighbours pointed out that new socio-economic activities (e.g. rural tourism, diving) were creating employment opportunities.

According to farmers and neighbours, food security is ensured by the availability of small family plots for subsistence crops (e.g. peas, wheat), livestock (e.g. pigs, sheep) and beekeeping (e.g. honey). In addition, access to fish (e.g. clams) and land resources (e.g. dates), a preference for local products (e.g. olive oil, goat’s cheese) and, especially in the past, the bartering of staple foods (e.g. eggs, bread) have enabled the inhabitants to live autonomously.

Water security for human consumption is covered by small springs. Access to water for irrigation, livestock and other uses is supported by flash-floods and *acequias* (traditional water transport ditches), rainfall and moisture from the sea. Interviewees emphasised the good quality of freshwater and its year-round availability. Water sufficiency during the long dry summer months is guaranteed by wells and *aljibes* (traditional water storage cisterns).

Interviewees expressed satisfaction when asked about their health status derived from the environment. They reported enjoying a balanced diet and mineral-medicinal water. They attributed their good physical condition to hiking, and their good mental condition to leisure in nature, enjoyment of the landscape and rural tourism, which give them peace and quiet.

Neighbours and farmers recounted how the uneven relief of the mountains and dry rivers provided shelter for war exiles, smugglers and peddlers to sleep, eat and trade goods (e.g. tobacco, cloth) and food (e.g. flour, oil). It was a place that was difficult to access and unknown to the security forces, even today to mass tourism, but offering its guests tranquillity and a milder climate than the surrounding area.

#### Beneficial NCP and GQL non-material dimensions

Nine GQL non-material dimensions promoted by beneficial NCP were identified through the code co-occurrence function: good social relationships, freedom of participation, freedom of choice and action, access to recreation and leisure, enjoyment of natural beauty, sense of cultural identity, access to knowledge and education, personal and physical security, and equity (Table 2, Fig. 4a). Interviewees reported having good social relationships with each other and described themselves as a supportive, respectful and trusting family. They attributed these values to the cooperation between natural and social systems. They recalled how women used to socialise while plaiting esparto, how farmers and shepherds worked together to graze and clear their land, and how neighbours cooperate in building crop terraces to protect the soil. They noted how hiking, diving and pilgrimages have fostered social links between locals and foreigners, and how, especially in the past, path through dry riverbeds encouraged meetings between young people and between traders.

According to the interviewees, the freedom to participate actively in society and in territorial decision-making is guaranteed by non-profit organisations (i.e. women's, retirees', socio-cultural, recreational and environmental associations), which are committed to sustainable development through the defence of heritage, cultural identity and local products. These associations convey the needs and feelings of social groups to the neighbourhood councils, which in turn communicate this information to the municipal council. The proactive attitude of the non-profit organisations has allowed some freedom of action and choice in the activities most valued by the social groups (e.g. traditional crops, pastoralism, hunting of small mammals, reforestation with native species, guided nature days). However, the interviewees expressed their continuing struggle against environmental legislation, which often overlooks their traditions, customs and heritage.

Interviewees expressed happiness and satisfaction at having access to multiple leisure activities, allowing them to spend more time with family and friends in a healthy way. They enjoy family picnics, hiking with cultural associations, farm work with grandparents, partridge shooting, identifying aromatic plants and playing in dry rivers. They noted that these are unique activities that they could not do elsewhere. Tourists reported enjoying the tranquillity and beauty of rural and beach tourism. Interviewees also reported feeling free and at ease when enjoying natural beauty (e.g. quiet and unpolluted landscapes, inspiring biodiversity).

Interviewees said they were proud to have grown up there and to have enjoyed its biodiversity (e.g. trees, partridges), jobs (e.g. farmer, goatherd), traditions (e.g. esparto plaiting, pilgrimages) and heritage (e.g. waterwheel wells, water mills), a sense of cultural identity that they would like to preserve and recover. They also proudly valued access to the knowledge of their ancestors (e.g. cultivation techniques, fishing gears), the access to an individualised and alternative school education that includes the value of nature, and environmental education projects (e.g. LIFE + project: *Astragalus nitidiflorus*).

In terms of personal and physical security, inhabitants from the upper and middle basins said they felt their families and belongings were safe from flooding, as dry rivers discharged floodwaters into the sea. They reported feeling protected in accessing resources (e.g. fish, grain, hiking) because there is no crime and the coastal geography facilitates their location. Finally, older interviewees referred to dry rivers and their resources as a public good for the enjoyment of all people. They emphasised the ability of their ancestors to distribute resources equitably.

### Relationships between detrimental NCP and GQL dimensions

**Fig. 5:**
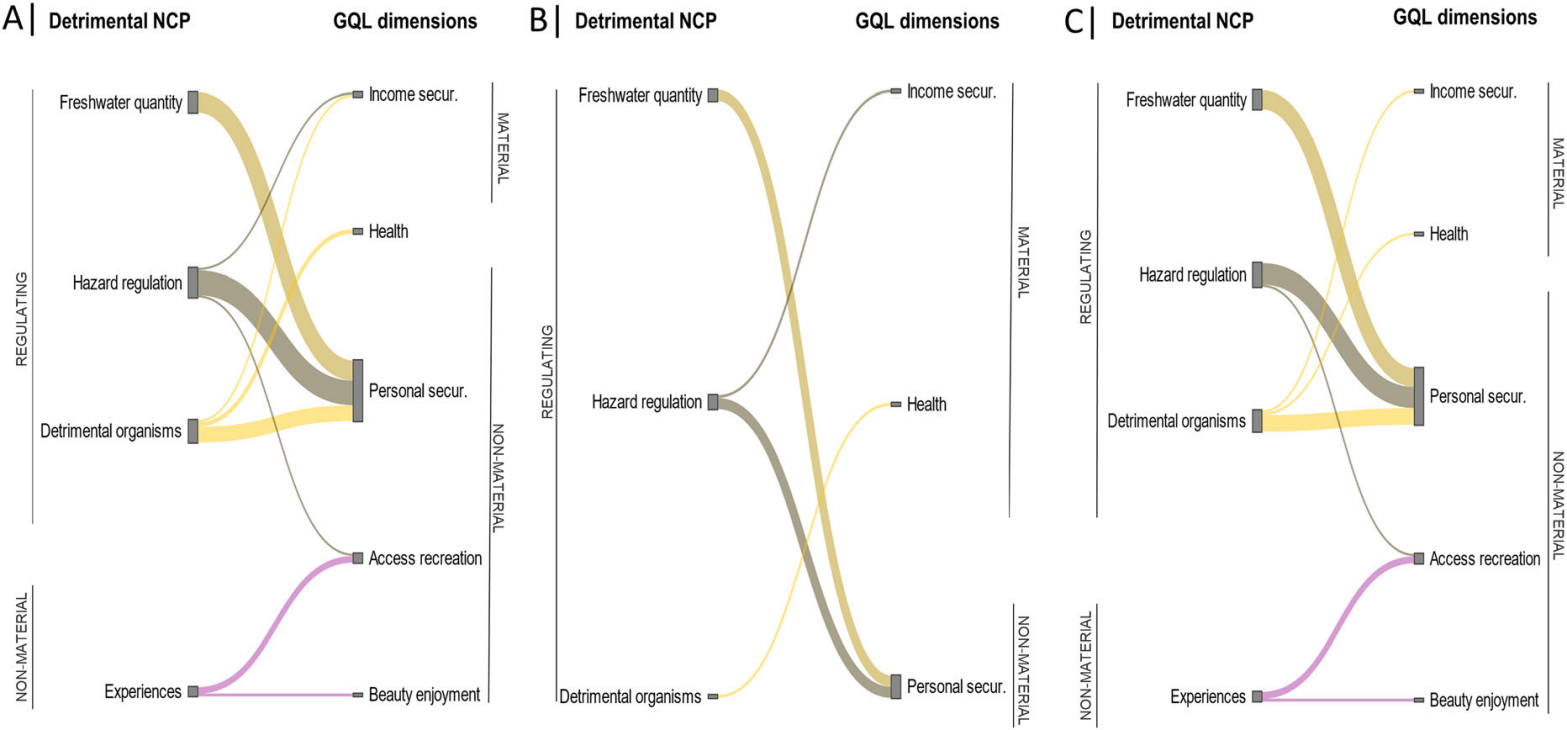
Relationships between detrimental NCP and GQL dimensions in the past and present (**a**), in the past only (**b**), and in the present only (**c**), as perceived by interviewees. The thickness of the lines indicates the number of interviewees who reported a relationship between a particular detrimental NCP and GQL dimension. The colours indicate the main NCP groups: regulating (yellow), non-material (pink)

From the code co-occurrence function, nine different relationships were identified between detrimental NCP and GQL dimensions, especially between regulating NCP and non-material dimensions (Fig. 5a). The relationship reported by the largest number of interviewees was regulation of hazards and extreme events with personal and physical security (*n* = 11) (Fig. S4). Five GQL dimensions were altered by detrimental NCP: personal and physical security, access to recreation and leisure, livelihood and income security, health, and enjoyment of natural beauty (Table 1).

According to the interviewees, personal and physical security was affected by large flash-floods from dry rivers and fires from dry reeds, grasses and pine trees. These hazards were described by neighbours as dangerous, causing damage to property (e.g. agricultural terraces, vehicles, dwellings) and people. Other minor detriments included small flash-floods destroying dry river paths and trapping and cutting off neighbours, wild boars damaging to agricultural terraces and gardens, and the uncontrolled growth of *Arundo donax* L. damaging traditional irrigation ditches and paths.

Some interviewees expressed dissatisfaction with access to recreation and leisure, and enjoyment of natural beauty. They dislike walking along dry rivers where overgrown vegetation hides the paths, and along beaches where there is seaweed. They also dislike some terrestrial plants, which they find ugly and dusty.

Drought was a concern for farmers as it affects traditional crops (e.g. almond, citrus and stone fruit trees) and native flora and fauna, threatening livelihood and income security. Other minor income security concerns were pests: whitefly, rabbits, noxious weeds and shrubs undermine crop growth; and foxes prey on goat herds. Farmers (due to soil loss), and water managers and scientists (due to loss of *Posidonia oceanica* (L.) DELILE and *Cymodocea nodosa* ASCH. meadows and fisheries) complained about large flash-floods, although none directly mentioned income insecurity.

In terms of health, interviewees complained about the lepidoptera *Thaumetopoea pityocampa* Denis & Schiffermüller, which defoliates pine trees and causes allergic reactions in animals and humans. Other pests mentioned were the borer coleopteran *Tomicus destruens* Woll. which feeds on pine trees, and the palm weevil which feeds on palm trees, although the latter two were not directly related to health.

### Influence of time scale on NCP-GQL relationships

On the one hand, the analysis of the relationships between beneficial NCP and GQL dimensions over time revealed that interviewees focussed on the relationships between material and regulating benefits and material GQL dimensions when describing past experiences, whereas they focussed on the relationships between non-material benefits and non-material GQL dimensions when describing present experiences (Figs. 4b, 4c, S2, S3). On the other hand, the analysis of the relationships between detrimental NCP and GQL dimensions over time revealed that while in the past GQL dimensions were affected only by regulating detriments, in the present they were also affected by non-material detriments (Figs. 5b, 5c, S5, S6).

### Influence of drivers of change on GQL

**Fig. 6:**
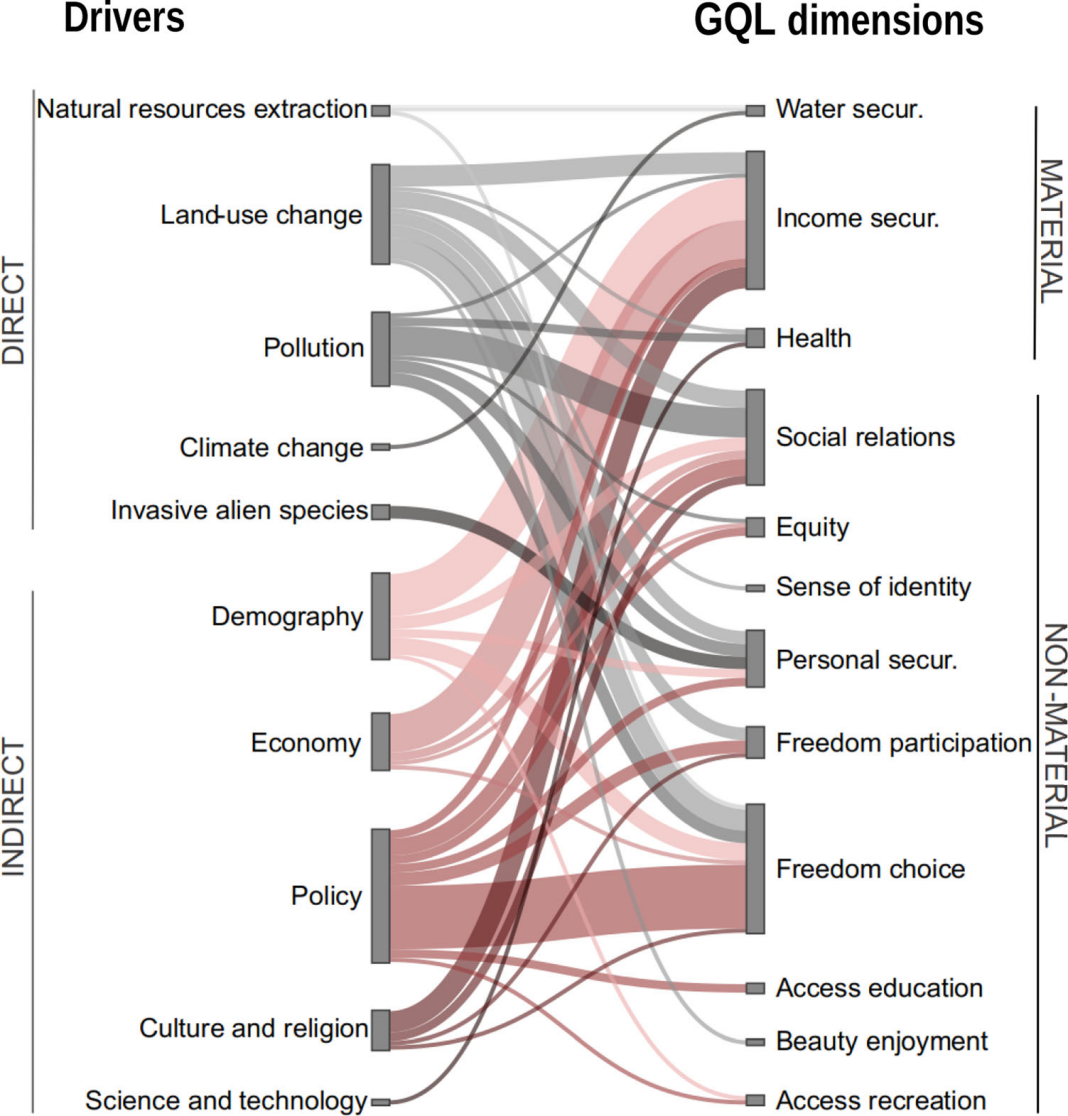
Relationships between drivers and GQL dimensions as perceived by interviewees. The thickness of the lines indicates the number of interviewees who reported a relationship between a particular driver and GQL dimension. The colours indicate the main groups of drivers: direct (grey) and indirect (red)

After coding the interviews, five direct and five indirect drivers were identified (Fig. 6). Forty different relationships were identified between the ten drivers and GQL dimensions (Fig. S7). The drivers that altered the highest number of GQL dimensions were policy (8 altered dimensions) and land-use change (8). The GQL dimension altered by the highest number of drivers was freedom of choice and action (7 drivers).

Farmers, hunters and fishermen expressed fear for their livelihood and income security as the production costs of local products (e.g. almonds) could not compete with the low prices of the international industrial market (economic driver). Moreover, local agricultural products are undermined by water insecurity due to overexploitation of aquifers (natural resource extraction) and droughts (climate change). Other local products such as vegetable fibres are being replaced by cheaper materials such as plastics (science and technology driver) (Table S7).

Most interviewees expressed that environmental policies of the past decades have restricted access to knowledge and education, freedom of participation, and freedom of choice and action. These policies have excluded most social groups, keeping them uninformed and deprived of their highly valued activities (e.g. building terraces, harvesting vegetable fibres) (Table S8). As a result, the relationship between managers and the rest of social groups has deteriorated. In this sense, the dumping of waste and overexploitation of freshwater by unregulated camper tourism and illegal settlements also undermined social relationships by affecting agriculture, livestock and fisheries.

The neighbourhood councils expressed inequity in the distribution of funds between rural development (scarce) and urban development (abundant). Similarly, farmers and neighbours expressed injustice at the low incomes earned from long days in the fields and the prohibition on expanding or renovating their dwellings for future generations due to economic globalisation and environmental policies respectively.

Other drivers included the replacement of former settlers, their traditional uses and cultural values with new ones such as tourists, motor sports, greenhouses and housing estates, which affect sense of cultural identity, enjoyment of natural beauty (e.g. greenhouses spoil the landscape), health (e.g. burning greenhouse pruning waste affects the respiratory system), personal and physical security (e.g. floodplain urbanisation increases flood risk), access to recreation (e.g. tourists crowd the beach) and livelihood and income security (e.g. disappearing traditional crops and grazing means economic loss).

## Discussion

### Integrating detriment co-production into research could raise social awareness and conservation of dry rivers

The generalising perspective of the IPBES framework classifies beneficial and detrimental NCP into 18 categories (Díaz et al. 2018). Recent reviews have demonstrated that dry rivers can provide 17 of the 18 categories (Steward et al. 2012; Koundouri et al. 2017; Datry et al. 2018; Vidal-Abarca et al. 2020, 2022; Nicolás-Ruiz et al. 2021). Additionally, a research conducted in the study area identified a further category related to the use of dry riverbeds for access to villages and fields (Nicolás-Ruiz et al. 2023). Nevertheless, none of the previous studies addressed the capacity of dry rivers to provide detriments. The present study demonstrates that dry rivers of the study area provide four detrimental NCP: regulation of freshwater quantity, location and timing (e.g. small floods), regulation of hazards and extreme events (e.g. large floods), regulation of detrimental organisms (e.g. animal attacks), and physical and psychological experiences (e.g. unpleasant walks) (Table 1).

The four NCP categories perceived as detrimental were also perceived as beneficial NCP (Figs. 4, 5). This occurs because elements and processes of ecosystems (e.g. an animal species, the water cycle) can be perceived negatively or positively (e.g. Pascual-Rico et al. 2020; Topp et al. 2022), depending on the value system of each individual (Pascual et al. 2017; Lliso et al. 2022). For instance, while some people enjoy the tranquillity and remoteness of dry rivers (Table 2), others find them unpleasant or boring (Table 1). This polarisation was also documented in a study that collected social perceptions from 30 people who were walking along the Negev Desert in Israel (Teff‐Seker and Orenstein 2019). Floodwaters were perceived as beneficial by inhabitants from upper basins (Table 2). In arid and semi-arid regions of southern Europe and western Asia, floodwaters are used for irrigation and livestock through ditches (García-Llorente et al. 2015), wells (Nasiri and Mafakheri 2015) and ponds (Alkhaddar 2003). However, inhabitants from lower basins considered floodwaters as detrimental, as they caused damage to facilities and fields (Table 1). This perception is frequently echoed by the mass media in the Mediterranean region (Rotger-Pujadas et al. 2023).

Most benefits of dry rivers are co-produced between natural and social systems (Vidal-Abarca et al. 2022; Nicolás-Ruiz et al. 2023). The descriptive analysis conducted in this study suggests that detriments may also be co-produced (Table 1). For instance, high property damage from flash-floods is associated with the drainage function of dry rivers and torrential rainfall, but increases when floodplains are urbanised (Quiñonero-Rubio et al. 2011; Pérez-Morales et al. 2016; Mazzoleni et al. 2022). Similarly, droughts increase the amount of dry biomass that fuels forest fires (Table 1). However, this risk increases even more if the social system abandons agroforestry thinning practices (Bravo de Guenni et al. 2005; Pereira et al. 2005). Many interviewees seemed unaware of the role that human activities play in providing benefits and detriments. Many of them did not associate infrastructure damage with floodplain urbanisation or increased pests and reduced rainfall with climate change, but with natural cycles. According to McMichael et al. (2005), these perceptions are common in urban and modernised areas, such as the coastal strip of the study area. Here, there is a dissociation between human daily needs and NCP, as many of these are replaced by anthropogenic goods (e.g. water supply, supermarkets). Co-production studies that integrate benefits and detriments can help raise awareness of the role that human activities play in the quality of life and conservation of dry rivers.

### GQL cannot be determined based solely on the potential of dry rivers to provide benefits

When asked about quality of life, interviewees referred to fourteen of the sixteen dimensions evaluated (Fig. 3). Only energy security and freedom to exercise spirituality were missing. Although energy security could be ensured by biomass-based fuels, such as brushwood, interviewees may have overlooked this option due to a greater preference for anthropogenic energy sources, such as electricity (Palomo et al. 2016). For example, Pereira et al. (2005) found that inhabitants of Sistelo (Portugal) replaced income and food from agricultural terraces and livestock with other jobs and imported goods. Conversely, fuelwood was being conserved because it was the only heating system available in most households. Freedom to exercise spirituality is supported by the benefit of supporting identities (e.g. pilgrimages along dry rivers) in Western cultures (Love et al. 2017; Martín-López et al. 2018). Nevertheless, interviewees associated this benefit with a sense of cultural identity and good social relationships, rather than with spirituality.

GQL dimensions were perceived in different ways depending on the interests of the interviewees. Some GQL dimensions were perceived mainly positively (e.g. food security), while others were perceived mainly negatively (e.g. personal and physical security). Other dimensions were perceived equally positively and negatively (e.g. good social relationships) (Fig. 3). These perceptions raise some interesting issues emerge that could be analysed in future studies. First, local food and recreation products seem to cover food security and access to recreation, but they do not seem to have an impact on livelihood and income security. Interviewees pointed to the low profitability of local products on the market. In Spain (Heider et al. 2021; Quintas-Soriano et al. 2023), as well as in other European and Asian regions (Subedi et al. 2022), traditional agriculture is struggling with low product prices and their high volatility due to growing industrial agriculture, as well as with rising costs of agricultural inputs and land management. For example, Nainggolan et al. (2012) found a trend towards low cereal and almond prices over the period 1976–2008 in semi-arid Mediterranean agroecosystems close to the study area.

Second, there seems to be freedom of participation, but not freedom of choice. Although interviewees reported mechanisms for social participation (e.g. neighbourhood councils, non-profit organisations), the interests and knowledge of some social groups may not be integrated in the decision-making processes. This may be due to different levels of influence and power between social groups regarding NCP management. For example, in a workshop comprising participants from diverse backgrounds and disciplines, Jorda-Capdevila et al. (2021) observed that the level of influence on NCP management in two non-perennial rivers of the Iberian Peninsula differed between social groups. This lack of choice would explain why social relationships were reported as both good (organisations and councils) and bad (administrations and institutions). Integrating local knowledge and interests into governance can provide nature-based solutions that contribute to biodiversity, benefits, quality of life, and resilience to disturbances such as climate change (Pereira et al. 2005; Palomo et al. 2021; Zvobgo et al. 2022). For example, floodwater harvesting systems such as *limans* in Israel (Paz-Kagan et al. 2017) or *qanats* in Iran (Nasiri and Mafakheri 2015) use infiltration processes and soil permeability to harvest freshwater and create afforestation groves. Finally, it is worth noting that personal and physical security was the GQL dimension that was perceived more negatively by interviewees and therefore may require more attention from management. This dimension was affected by many detriments (e.g. large flash-floods, fires, animal attacks; Fig. 5), which are increasing due to land-use change, invasive alien species and policies, among other drivers (Fig. 6).

Most studies assume that the potential of dry rivers to provide benefits leads to a GQL. However, the dry rivers of the study area, while providing multiple benefits, do not support all the dimensions that interviewees identified as necessary to achieve a GQL. This occurs because some benefits are reduced and some detriments are increased by drivers of change. For example, flash floods are perceived as a benefit in the upper basins, contributing to water security, and as a detriment in the lower basins, affecting human security. It is thus essential to identify and assess the dimensions that, according to each social system, contribute to a fulfilled life in order to ascertain whether a GQL is actually enjoyed, regardless of the potential benefits that dry rivers may provide. The status of the different GQL dimensions can also provide insights into the functioning of the social-ecological system, which could inform management strategies. Given the diversity of perceptions observed, dialogue between social groups is essential to reach agreements and implement inclusive policies. Finally, it would be beneficial for future research to consider the possibility that different dimensions of GQL may not be considered equally important by social systems.

### Relationships between NCP categories and GQL dimensions

The code co-occurrence function between NCP categories and GQL dimensions revealed that five GQL dimensions were influenced by both beneficial and detrimental NCP (Figs. 4a, 5a). Some of these dimensions were perceived positively by interviewees and others negatively (Fig. 3). This suggests that the influence of benefits and detriments on these dimensions may not be balanced. Future research should assess the resulting impact that benefits and detriments have on each GQL dimension in order to avoid misinterpretation of quality of life and to facilitate the development of effective management measures.

Most of the relationships between NCP categories and GQL dimensions were not one-to-one, but many-to-many (Figs. 4, 5). This indicates that one NCP can influence several GQL dimensions. From a management perspective, this suggests that it may be necessary to act on a set of NCP, rather than just one, in order to restore one altered GQL dimension. For example, income security depends on eight benefits, which must be promoted together to achieve a GQL. In those dry rivers where many GQL dimensions are altered, it will generally be more appropriate to implement those measures that promote a higher diversity of benefits and a lower diversity of detriments.

The present study demonstrates that the strong connection of local communities to the natural system of the study area is associated with a positive perception of dry rivers as providers of benefits that contribute to a GQL, confirming the first hypothesis. In contrast, previous studies on social perception, where not all social representatives had a connection to the natural system, showed a more limited perception of the capacity of dry rivers to contribute to GQL (García-Llorente et al. 2015; Leigh et al. 2019; Rodríguez-Lozano et al. 2020).

The results of the temporal analysis suggest that the relationships between NCP and GQL may have been more diverse in the past than in the present, as relationships between non-material benefits and non-material dimensions seem to be more strongly perceived at present (Figs. 4b, 4c). Martínez and Martínez-Carrasco (2019) analysed the socio-territorial processes of the study area through a literature review and local interviews, and found a trend of increasing recreational activities and GQL non-material dimensions to the detriment of traditional farming activities and material GQL dimensions. They called this perception change landscape musealisation and attributed it to political, economic and socio-cultural factors. For example, the protection status assigned to the study area over the last 30 years (e.g. Protected Natural Area, Special Protection Area, Site of Community Importance), has led to the implementation of restrictive environmental policies that have limited traditional socio-economic activities (Martínez and Martínez-Carrasco 2019). A study of the capability of protected areas to provide benefits in the semi-arid region of Spain associated high levels of restriction with a reduction in material (e.g. food) and regulating benefits (e.g. freshwater regulation) (Castro et al. 2015). The accessibility and social perception of these two types of benefits are also negatively affected by the low profitability of local products, as discussed in the previous section.

The observed change in social perception is also related to socio-cultural factors, including the rural exodus and the arrival of new settlers. Rural exodus and land abandonment caused by restrictive policies and low incomes lead to the loss of local ecological knowledge, values and beliefs related to the maintenance of farming, traditional floodwater harvesting systems and raw materials (Quintas-Soriano et al. 2016, 2022, 2023). At the same time, the arrival of new settlers increases the value of access to recreation and leisure (e.g. hiking, family picnics) and the enjoyment of natural beauty (e.g. unpolluted landscapes, inspiring wildlife). These settlers seek a quiet place to live and enjoy leisure, but are rarely involved in traditional farming (Martínez and Martínez-Carrasco 2019). The most recent census conducted by the Cartagena City Council reveals a threefold increase in the population of the study area over the past 25 years. In particular, foreign population has increased markedly, from 6% of the total population in 2000 to 33% in 2024 (AdC 2021).

In addition to the reduction in benefits, the process of landscape musealisation may also be contributing to an increase in detriments. For example, the expansion of urban areas along dry rivers in the coastal strip has increased by 12% in recent decades, according to the data viewer of the Copernicus Land Monitoring Service of the European Union.^2^ This process of urbanisation has already been identified as an increasing risk factor for floods in the surrounding area of the study area (Quiñonero-Rubio et al. 2011; Pérez-Morales et al. 2016). The increase in unpleasant experiences may be related to the loss of local knowledge regarding the management of animal and plant species (e.g. plants taking over paths).

The observed changes in the study area provide empirical support for the second hypothesis of this study. Drivers of change reduce benefits and increase detriments, influencing the social perceptions of dry rivers. This is consistent with the changes in relationships between NCP and GQL in space and time observed by Liu et al. (2023) in four dryland areas in Mongolia. It is therefore evident that the management of dry rivers must be adapted to each specific spatio-temporal context.

### Limitations and methodological considerations

The detrimental NCP categories, GQL dimensions and types of drivers of change identified in this study, as well as the beneficial NCP categories identified in the preliminary study, are highly representative of the social system of the study area. The saturation point for the aforementioned variables was reached during the course of the interviews. The saturation point can be defined as the number of interviews from which no new information related to the study objectives is found (Knott et al. 2022). To ascertain this, the NCP categories, GQL dimensions and drivers of change were listed in an informal way (i.e. without coding) after each interview, until no new issues emerged or did so very slowly (i.e. many interviews were required). This means that the existence of some additional topics related to the study objectives is not precluded, but that they were not included due to time and funding constraints.

The relationships between NCP categories, GQL dimensions and drivers of change, as well as their changes over time, may be unrepresentative of the social system, as their saturation point was not considered. This implies that other interactions, which are not evidenced by this paper, could exist. However, the aim of this study is not to identify all these interactions; rather, it is to demonstrate that they exist and to identify general factors that can be useful for a GQL management. With regard to the quantitative data, the number of interviewees who perceived the different NCP categories, GQL dimensions and drivers of change, as well as the number of interviewees who reported relationships between these variables, should only be used to formulate research hypotheses due to the limited sample size.

Snowball sampling has been criticised because participants may have similar interests and perceptions to the initial interviewee. To minimise this bias and ensure the greatest possible diversity of perceptions, we screened potential participants by asking them about their socio-cultural and socio-economic data. In addition, the sample was developed around three initial interviewees (two participants from the neighbourhood council group and one participant from the manager group). Finally, gender bias could be present in the study, as women (*n* = 9) were less represented than men (*n* = 28). This could be explained by the fact that the intensification and mechanisation of primary production, coupled with outmigration through the acquisition of higher-level qualifications, has led to a situation where field work is dominated by men (Iniesta-Arandia et al. 2015). Another reason is due to that women have had fewer opportunities to reach high positions in companies, organisations and academia (Sánchez-Montoya et al. 2016; Catalán et al. 2023), the positions used in this study.

## Conclusions

The social perception of the contribution of dry rivers to a GQL varies between communities. Local communities whose livelihoods and knowledge are closely linked to nature perceive the benefits of dry rivers as contributing to a GQL, in contrast to other communities that are not so closely linked. These benefits contribute to multiple and different GQL dimensions. The implementation of management strategies that facilitate the provision of a diverse range of benefits is likely to have a significant impact on GQL. Natural and social systems of dry rivers co-produce not only benefits (e.g. freshwater harvesting systems) but also detriments (e.g. flash-floods, fires, pests). Integrating the co-production of detriments and benefits into research can raise people’s awareness of the role that human activities play in maintaining GQL. Drivers of change such as economic globalisation, restrictive environmental policies and rural depopulation affect GQL, increasing detriments and reducing benefits. These changes in turn contribute to a pejorative perception of dry rivers. The different social perceptions of dry rivers among communities and individuals over time imply that the management of these ecosystems must be adapted to each specific spatio-temporal context. This adaptation requires the development of social methodologies that promote citizen participation, the achievement of agreements between social groups, and the implementation of integrative management strategies to achieve a GQL.

## Supplementary Information

Below is the link to the electronic supplementary material.

Supplementary file1 (PDF 865 KB)

## Footnotes

1 https://sankeymatic.com/

2 https://land.copernicus.eu.
