# Supplementary material for: Can dry rivers provide a good quality of life? Integrating beneficial and detrimental nature’s contributions to people over time
Source: Ambio. 2024 Sep 24;54(2):305–24. doi: 10.1007/s13280-024-02072-x (PMC11662126; doi:10.1007/s13280-024-02072-x)
Supplement: Supplementary file 1 — Supplementary file1 (PDF 865 KB) [file 13280_2024_2072_MOESM1_ESM.pdf]

Ambio

Supplementary Information

This supplementary information has not been peer reviewed

Title: **Can dry rivers provide a good quality of life? Integrating beneficial and detrimental nature's contributions to people over time**

Table S1

Ten of the most influential social groups in the study area. The name (social group), abbreviation, number of representatives (n) and description of each social group are given

Social group	Abbreviation	n	Description
Neighbours	NEI	7	People with socio-cultural, environmental or other interests related to dry rivers who do not belong to any other social group. It includes different professional profiles: secondary school teachers, language teachers, gym instructors, administrative staff, etc.
Non-profit organisations	ORG	6	A group of people who altruistically pursue the same cultural, social or environmental goal in the study area
Environmental managers	MAN	5	An official engaged in the planning, management and protection of the natural environment through the regulation of legislation
Crop farmers	CRO	4	A person whose main occupation is agriculture. This includes traditional rainfed agriculture, small irrigated orchards and large greenhouses
Researchers	RES	4	University teaching and research staff
Tourism sector	TOU	3	A person who works in the tourism sector. This includes ecotourism, camping and diving in the marine area near the dry rivers
Hunters	HUN	2	A person who hunts small mammals and birds for self-consumption or as a leisure activity
Fishermen	FIS	2	A person engaged in traditional fishing gear in the marine area around dry rivers
Livestock farmers	LIV	2	A person whose main occupation is animal husbandry. This includes traditional pastoralism and livestock farming
Neighbourhood councils	COU	2	Local decision-making bodies with a political character that depend on the municipal government

Table S2

Representatives of the social groups interviewed and their main socio-economic data. For each representative, the following information is provided: identification code (code), social group to which he/she belongs (type, subtype), professional position (position), gender, age, basin to which he/she belongs (basin), part of the basin where he/she operates (part) and level of education (education)

Code	Type	Subtype	Position	Gender	Age	Basin	Part	Education
1_CRO_T1	crop farmer	extensive rainfed (traditional)	self-employed worker	M	74	Azohía	upper	primary school
1_CRO_T2	crop farmer	extensive rainfed (traditional)	self-employed worker	M	50	Cañar	upper	secondary school
1_CRO_T3	crop farmer	extensive rainfed (traditional)	self-employed worker	M	74	Cañar	upper	*NA*
1_CRO_I1	crop farmer	intensive irrigated (industrial)	spokeswoman of private company	F	45	Cañar	lower	university
2_MAN_L1	environmental manager	land	spokesman of the regional environment department	M	48	Valdelentisco, Cañar, Azohía	upper, lower	university
2_MAN_L2	environmental manager	land	spokesman of the regional environment department	M	36	Valdelentisco, Cañar, Azohía	upper, lower	university
2_MAN_W1	environmental manager	water	CEO of the purification plant	F	28	Cañar	lower	university
2_MAN_W2	environmental manager	water	Spokesman of dry river basin authority	M	62	Valdelentisco, Cañar, Azohía	upper, lower	university
2_MAN_W3	environmental manager	water	CEO of the desalination plant	M	43	Valdelentisco	lower	university
3_NEI_A1	neighbour	from abroad*	ordinary citizen	F	51	Cañar	lower	secondary school
3_NEI_A2	neighbour	from abroad**	ordinary citizen	F	52	Cañar	lower	secondary school
3_NEI_B1	neighbour	from basin	ordinary citizen	M	28	Valdelentisco, Cañar	upper	university
3_NEI_B2	neighbour	from basin	ordinary citizen	F	37	Cañar, Azohía	upper	secondary school
3_NEI_B3	neighbour	from basin	ordinary citizen	M	54	Cañar	upper	university
3_NEI_R1	neighbour	from region	ordinary citizen	F	57	Cañar	upper	secondary school
3_NEI_R2	neighbour	from region	ordinary citizen	M	50	Valdelentisco	upper	secondary school
4_HUN_1	hunter	traditional hunter	spokesman of hunting society	M	86	Cañar, Azohía	upper	primary school
4_HUN_2	hunter	recreational hunter	chairman of hunting society	M	51	Valdelentisco	upper	secondary school
5_RES_1	researcher	ecologist	academic research staff at University of Murcia	M	55	Azohía	lower	university
5_RES_2	researcher	ecologist	academic research staff at University of Murcia	M	61	Valdelentisco, Cañar, Azohía	upper	university
5_RES_3	researcher	botanist	academic research staff at University of Murcia	M	62	Valdelentisco	upper	university
5_RES_4	researcher	botanist	academic research staff at University of Murcia	M	63	Cañar, Azohía	upper	university
6_TOU_1	tourism sector	campsite	CEO of private company	M	69	Valdelentisco, Cañar	upper, lower	secondary school
6_TOU_2	tourism sector	ecotourism	CEO of private company	M	55	Azohía	upper	university
6_TOU_3	tourism sector	diving	Spokeswoman of private company	F	49	Cañar, Azohía	lower	secondary school
7_LIV_T1	livestock farmer	traditional goatherd	self-employed worker	M	47	Azohía	upper	primary school
7_LIV_I1	livestock farmer	industrial livestock farmer	self-employed worker	M	59	Valdelentisco, Cañar	upper	secondary school
8_ORG_C1	non-profit organisation	cultural	chairman	M	80	Cañar, Azohía	upper, lower	university
8_ORG_C2	non-profit organisation	cultural	chairman	M	*NA*	Cañar, Azohía	upper, lower	university
8_ORG_E1	non-profit organisation	environmental	chairman	M	60	Azohía	lower	university
8_ORG_E2	non-profit organisation	environmental	chairman	M	55	Cañar	upper	university
8_ORG_S1	non-profit organisation	social	spokeswoman of women association	F	64	Azohía	lower	secondary school
8_ORG_S2	non-profit organisation	social	spokesman of senior association	M	70	Azohía	lower	secondary school
9_FIS_1	fisherman	traditional	self-employed worker	M	73	Azohía	lower	secondary school
9_FIS_2	fisherman	traditional	CEO of private company	M	57	Azohía	lower	*NA*
10_COU_1	neighbourhood council	upper part	chairman	F	43	Cañar, Azohía	upper	secondary school
10_COU_2	neighbourhood council	lower part	chairman	M	64	Valdelentisco, Azohía	lower	university

CRO_T: traditional crop farmer, **CRO_I**: industrial crop farmer, **MAN_L**: land environmental manager, **MAN_W**: water environmental manager, **NEI_A**: neighbour born abroad, **NEI_B**: basin-born neighbour, **NEI_R**: region-born neighbour, **HUN**: hunter, **RES**: researcher, **TOU**: tourism sector, **LIV_T**: traditional livestock farmer, **LIV_I**: industrial livestock farmer, **ORG_C**: cultural non-profit organisation, **ORG_E**: environmental non-profit organisation, **ORG_S**: social non-profit organisation, **FIS:** fisherman, **COU**: neighbourhood council; **CEO**: Chief Executive Officer; **M**: male, **F**: female; ***NA*** : not available; *****: 18 years living in the study area; ******: 15 years living in the study area

Table S3

Questionnaire for the analysis of the relationships between the natural system of dry rivers and the associated social system. The questionnaire was structured around five topics, each of which included a series of questions linked to different objectives. The objectives were designed to be addressed by three separate studies. The initial study, which has already been published (Nicolás-Ruiz et al. 2023), addressed dry river benefits, their synergies and trade-offs (objective 1). The current study addressed dry river detriments, quality of life, and, to a lesser extent, drivers of change (objective 2). A subsequent study will examine the impact of drivers of change on the benefits and detriments of dry rivers (objective 3)

Topics	Questions	Objectives
Benefits	What benefits do dry rivers provide to you?<br>What activities do you do in dry rivers?<br>What do you use dry rivers for?	1a
Detriments	Do dry rivers harm you in anyway?	2a
Quality of life	Are you happy here?<br>How you rate your quality of life?	2b
	How do benefits of dry rivers contribute your well-being?<br>What aspects of your well-being do dry rivers fulfil?	2b, 2c
	How do detriments of dry rivers affect your well-being?<br>What aspects of your well-being are affected by these harms?	2b, 2c
Conflicts	Is there conflict or cooperation between different social groups?<br>Is there conflict or cooperation in relation to the environment?	1b, 3b, 3c
	Do you think your activity benefits or harms other social groups?	1b, 3b, 3c
Drivers of change	Have you noticed any changes in relation to dry rivers in recent decades?<br>Have the benefits and detriments changed over last decades?	2d, 3a, 3b
	What do you think are the reasons for these changes?<br>Who other factor or social actors have involved?	2d, 3a, 3b
	How have these changes affected your well-being?	2d

First study. Co-production of nature's contributions to people in dry rivers: a case study in Murcia, Spain (Nicolás-Ruiz et al. 2023). General objective: to assess the capacity of dry rivers to provide benefits. Specific objectives: to analyse the processes of benefit co-production between dry rivers and the associated social systems (1a), and to determine how benefit co-production influences the development of synergies and trade-offs (1b).

Current study. Can dry rivers provide a good quality of life? Integrating beneficial and detrimental nature's contributions to people over time. General objective: to analyse the capacity of dry rivers to provide a good quality of life. Specific objectives: to identify detriments of dry rivers (2a), to characterise how the social system of dry rivers perceives a good quality of life (2b), to determine the relationships between benefits and detriments of dry rivers and the dimensions of a good quality of life (2c), and to assess changes in social perceptions of a good quality of life over time (2d).

Future study. Impact of drivers of change on nature's contributions to people in dry rivers. General objective: to characterise the drivers of change affecting nature's contributions to people in dry rivers. Specific objectives: to identify the direct and indirect drivers affecting dry rivers (3a), to identify the relationships between drivers of change and nature's contributions to people (3b), and to identify the relationships between direct and indirect drivers of change (3c).

Table S4

Categories of beneficial and detrimental Nature's Contributions to People (NCP), proposed by the generalising perspective of the IPBES conceptual framework (Díaz et al. 2018). The definitions of the categories were adapted as much as possible to the dry river context. The beneficial NCP categories perceived by the interviewees in the study area were analysed in a preliminary study (Nicolás-Ruiz et al. 2023) and are marked with an X

NCP		Definition	Nicolás-Ruiz et al. 2023
Regulating:			
1	Habitat creation and maintenance	Production by dry rivers and their biodiversity of the ecological conditions necessary for living beings of importance to humans (e.g., growing sites for crops, roosting places for agricultural pests)	X
2	Pollination and dispersal of seeds	Pollination of plants and dispersal of seeds of importance to humans by animals and wind. This includes the harmful effects that pollen and spores can have on humans	X*
3	Regulation of air quality	Regulation by ecosystems of CO_2/O_2 balance, nitrogen oxides, allergens and other pollutants that affect human health or infrastructures	X
4	Regulation of climate	Regulation of temperature and humidity by vegetation cover, and regulation of greenhouse gas emissions by dry riverbeds (e.g., organic carbon storage)	X
5	Regulation of ocean acidification	Regulation of CO_2 and seawater pH, which affect marine organisms important to humans (e.g., corals)	-
6	Regulation of freshwater quantity, location and timing	Regulation of surface and groundwater that positively (e.g., freshwater-harvesting systems, traditional ditches, wells, springs) and negatively (e.g., erosion of arable land, social and communication isolation due to flooding) affect people	X
7	Regulation of freshwater quality	Regulation of the physical, chemical and biological properties of freshwater (e.g., microbial denitrification, filtration by aquifers and springs)	X
8	Formation and protection of soils	Protection of soil from erosion and processes that underlie soil fertility by plants, soil organisms and humans (e.g., crop terraces prevent erosion). It includes the filtration and storage of chemical and biological pollutants (e.g., nutrients, pathogens) in soils	X
9	Regulation of hazards and extreme events	Regulation of extreme natural events by ecosystems by reducing or increasing their intensity and frequency (e.g., floods, storms, fires, landslides)	X
10	Regulation of detrimental organisms	Positive effects of pest and predator control that affect humans and their interest species and habitats by organisms. It also includes negative effects of organisms on humans (e.g., animal attacks), their plants (e.g., pests), animals (e.g., parasites) or infrastructure (e.g., damage by pigeons, termites, roots)	X
Material:			
11	Energy	Production of biomass-based fuels such as biofuel crops, animal waste and fuelwood	X
12	Food and feed	Production of food by wild and human-managed organisms (e.g., rabbits, cereal crops) and feed for domesticated animals (e.g., goats, sheep)	X
13	Materials, companionship and labour	Production of materials derived from biotic (e.g., vegetable fibres) and abiotic elements (e.g., sands). It includes the availability of pets and labour animals	X
14	Medicinal and genetic resources	Production of medicinal materials derived from organisms (e.g., plants), and animal breeds and plant varieties of importance for humans	X
Non-material:			
15	Learning and inspiration	Provision by dry rivers of opportunities to gain traditional knowledge and inspiration for art and technological design	X
16	Physical and psychological experiences	Provision by dry rivers of opportunities to develop physical (e.g., hiking, tourism) and psychological activities (e.g., relaxation, aesthetic enjoyment). These activities may be seen as beneficial or detrimental depending on the culture of individuals and social groups	X
17	Supporting identities	Provision by ecosystems of a sense of belonging and connectedness that underpins narratives and celebrations. It includes the satisfaction (or fear) of knowing that a landscape or species exists	X
Other:			
18	Maintenance of options	Capacity of dry rivers to provide future regulating, material and non-material benefits (or detriments) derived from conservation and evolution of species (e.g., new medicines)	X
19	Access to villages and fields	Dry riverbeds are used as a link between isolated villages, places of interest, houses, individuals, etc., promoting good social relationships, local trade and local leisure. This is the only category that is not directly considered in the generalising perspective of the IPBES framework. However, it is well documented in the literature	X

* Pollination and dispersal of seed was overlooked by interviewees despite the fact that dry river vegetation supports pollinators and family beekeeping has been traditionally practised according to the literature

Table S5

Material and non-material dimensions of a Good Quality of Life (GQL) used as categories for coding the interviews, as proposed in the Global Assessment Report on Biodiversity and Ecosystem Services developed by the Intergovernmental Science-Policy Platform on Biodiversity and Ecosystem Services (IPBES). Adapted from Brondízio et al. (2019) for the dry river context (Vidal-Abarca et al. 2022; Nicolás-Ruiz et al. 2023)

GQL dimensions		Description
Material:		
1	Food security	Availability, access and preference for local products (e.g., olive oil, milk goat, carob flour)
2	Water security	Availability and access to sufficient quality freshwater (e.g., access to quality borehole water for goat hydration)
3	Energy security	Energy availability, access and affordability (e.g., access to brushwood, solar energy)
4	Shelter	Ability to provide a clean and safe refuge for resting, feeding, hiding or protecting from natural (e.g., heat and cold waves) or anthropogenic hazards (e.g., wars). For example, war exiles and smugglers find refuge in dry rivers
5	Livelihood and income security	Ability to access natural resources and income necessary to fulfil material needs and social obligations. It includes work opportunities (e.g., generate sufficient income to make a living from farming, beekeeping, collecting plant fibres, etc.)
6	Health	Ability to enjoy physical and psychological health (e.g., staying free from stress and pollution)
Non-material:		
7	Good social relationships	Ability to provide assistance, support and mutual understanding between different individuals and social groups. It includes social cohesion, mutual respect, good family relations, etc. (e.g., cooperation between farmers and herders at work)
8	Equity	Parity status at the level of gender, age, race, income, social class, etc. (e.g., equitable distribution of natural resources across social classes)
9	Sense of cultural identity	Sense of belonging to a social group linked to a locality, ethnicity, activity, gender, etc. It involves the development of activities that are culturally valued or linked to intangible values (e.g., satisfaction from collecting and plaiting esparto grass)
10	Personal and physical security	Secure access to natural resources, safety of person and possessions (e.g., dry rivers drain rainwater and prevent human and material damage from flooding)
11	Freedom of participation	Access to opportunities for active participation in society. This includes participation in electoral processes, budget processes, non-profit organisations, land stewardship, etc. (e.g., territorial decision-making involves neighbourhood associations). This dimension is combined with that of good social relations in the IPBES global report
12	Freedom of choice and action	Having control over what a person values doing or being (e.g., freedom to harvest wild fruits, medicinal herbs and plant fibres such as esparto grass)
13	Access to knowledge and education	Access to education, knowledge, learning new skills and information necessary to participate in society and purse culturally valued aspirations (e.g., access to learning about traditional freshwater harvesting systems)
14	Freedom to exercise spirituality	Freedom to exercise one's faith, beliefs and religious practices (e.g., pilgrimages, sacred natural sites)
15	Access to recreation and leisure	Access to socially valued leisure activities that provide satisfaction or pleasure. They are often associated with improving health and spending quality time with friends and family (e.g., family picnics, rural tourism)
16	Enjoyment of natural beauty	Ability to enjoy the beauty of nature aesthetically, acoustically, as a source of inspiration, or as a place for reflection, meditation or comfort (e.g., enjoy silent and unpolluted landscapes)

Table S6

Direct and indirect drivers of change used as categories for coding the interviews, as proposed in the Regional Assessment Report on Biodiversity and Ecosystem Services for Europe and Central Asia developed by the Intergovernmental Science-Policy Platform on Biodiversity and Ecosystem Services (IPBES). Adapted from Elbakidze et al. (2018) for the dry river context (Vidal-Abarca et al., 2020, 2022; Nicolás-Ruiz et al., 2021)

Drivers of change		Description
Direct:		
1	Natural resources extraction	Overexploitation of abiotic (e.g., groundwater, sand, gravel) and biotic (e.g., medicinal herbs, vegetable fibres, fish and seafood whose habitat is the mouth of the dry river, hunting animals) resources
2	Land-use change	Changes in agricultural systems (e.g., from extensive to intensive, from rainfed to irrigated, greenhouse construction), traditional land uses (e.g., traditional pastoralism disappearing, land abandonment), urban development (e.g., construction of housing estates, dams, roads) and protected areas (e.g., conserving of wildlife by restricting traditional activities)
3	Pollution	Pollution of dry riverbeds and groundwater by nutrients (nitrogen and phosphorus from agriculture, urban wastewater), heavy metals from mines and quarries, excessive lighting and noise, rubbish, plastics, construction waste
4	Climate change	Changes in climatic conditions. For example, increase in temperature, decrease in precipitation, rise in sea level, increase in extreme events (e.g., droughts, exceptional flash-floods), decrease in freshwater reserves (e.g., groundwater, springs, dry river flow) and increase in desertification processes
5	Invasive alien species	Introduction of invasive alien species, both plants and animals, through accidental introduction (e.g., insects hidden in goods, escaped animals), ornamental use (e.g., cacti, flowering plants) and economic factors (e.g., fast-growing fodder plants, high-yielding crop varieties)
Indirect:		
6	Demographic	Demographic factors relate to population density and growth (e.g., depopulation, mass tourism), migration (e.g., rural exodus to cities due to lack of employment opportunities, return to rural areas due to wars and conflicts) and population ageing due to low birth rates
7	Economy	Changes in economic models (e.g., from local sustainable economies to global trade). Global trade influences in production and consumption patterns, accessibility and prices of products, taxes and subsidies, and fiscal reforms. These factors have a particular impact on rural communities that are not integrated into the global economy
8	Institutional or policy	Factors related to governance models and legislation that provide formal institutions for natural resource management (e.g., regulations governing agriculture, mining, forestry and tourism, integration of environmental policies). It includes the institutional capacity to regulate the private and public sectors, and the existence of political or armed conflicts.
9	Culture and religion	The values, beliefs and social norms provided by cultural and other social groups (e.g., nations, ethnic groups, professions, organisations, gender, worldviews) influence the way people relate and act with nature, often manifested through lifestyles and consumption patterns (e.g., veganism, sedentarisation of nomadic pastoralists).
10	Scientific and technological	Technological innovations influence the way natural resources are managed (e.g., technologies of information and communication, food, materials, industry; artificial intelligence) influence agricultural intensification, economic growth, etc.

Table S7

Material dimensions of a good quality of life (GQL) affected by the drivers of change in the dry rivers of the study area. The type and number of drivers (N) affecting each GQL dimension are indicated. The relationships between drivers and GQL dimensions are exemplified by verbatim quotes from the interviewees

Material dimensions	Drivers of change			Examples	
	**Direct**	**Indirect**	**N**	**Verbatim quotes**	**Interviewee**
Livelihood and income security	pollution, land-use change	demography, economy, policy, culture and religion	6	"Crops are no longer profitable because it costs a lot of money to maintain them. For example, almonds are now worth twice less because of competition from almonds from abroad" **[economy]**	1_CRO_T1
				"I am very angry about the frozen and gutted fish coming in from overseas because it drives down the price of our fresh fish" **[economy]**	9_FIS_2
Health	pollution, land-use change	science and technology	3	"The greenhouses sometimes burn their waste four or five times a year: roots, pesticides, plastics. We can't open the windows because the smoke is very toxic" **[pollution]**	3_NEI_A2
				"The children can't sometimes go out for recess because of the smoke from the burning greenhouses" **[pollution]**	3_NEI_A1
Water security	natural resources extraction, climate change	*na*	2	"I notice that it is raining much less than before and the water is running out. Whoever thinks there will be water in the future is wrong" **[climate change]**	6_TOU_1
				"Modern wells are more powerful and take water from dry river wells and subsistence springs, which are disappearing" **[natural resources extraction]**	2_MAN_W2

CRO_T: traditional crop farmer, **FIS:** fisherman, **NEI_A:** foreign-born neighbour, **TOU:** tourism sector, **MAN_W:** water environmental manager; ***na***: not applicable

Table S8

Non-material dimensions of a good quality of life (GQL) affected by the drivers of change in dry rivers of the study area. The type and number of drivers (N) affecting each GQL dimension are indicated. The relationships between drivers and GQL dimensions are exemplified by verbatim quotes from the interviewees

Non-material dimensions	Drivers of change			Examples	
	Direct	Indirect	N	Verbatim quotes	Interviewee
Freedom of choice and action	natural resources extraction, pollution, land-use change	demography, economy, policy, culture and religion	7	"When they introduced it [new environmental laws] in 2001, it was very bad for us because they didn't consider that we wanted to stay here, in the countryside. They wouldn't even let you plough the land" **[policy, land-use change]**	10_COU_1
				"There are fewer and fewer people in upland areas because the policy of protected areas tends to exclude people from their natural spaces, and there are no longer people who have internalised this space as part of their live" **[policy, demography]**	5_RES_3
Good social relationships	pollution, land-use change	demography, economy, policy, culture and religion	6	"We have problems with campers because they camp their caravans everywhere leaving rubbish behind" (3_NEI-B3) **[pollution]**	3_NEI_B3
				"The biggest social conflict has been urban sprawl, although the crisis [Spanish property bubble] has greatly reduce it" **[land-use change]**	8_ORG_E2
Personal and physical security	invasive alien species, pollution, land-use change	demography, policy	5	"In the past, the cane [*Arundo donax*] was used for tomato plants, fishing, brooms... but not now and it has invaded the dry riverbeds. We don't want to remove them, but we do want to control them so that they don't collapse the riverbed and flood nearby houses" **[invasive alien species]**	10_COU_1
				"People are throwing debris and rubbish into dry rivers and the riverbeds are narrowing. The day there is a flash flood you will see the problems it causes" **[pollution]**	8_ORG_S2
Equity	pollution	economy, policy	3	"I have fought hard for welfare, but they [politicians] have taken away our collective agreement. The working conditions in the countryside are disgraceful, 2-hour contracts working 12 hours" **[policy]**	1_CRO_T1
				"We are the forgotten villages of Cartagena and its dry rivers too. They are impassable and to repair them we have to fight the city council and the Segura Hydrographic Confederation [river basin management body]" **[policy]**	10_COU_2
Freedom of participation	land-use change	policy, culture and religion	3	"When it came to creating the protected area, we had no contact with the regional environment department. They didn't ask us anything, they excluded us" **[land-use change]**	1_CRO_I1
				"The urban growth of the rural areas in the protected area was not taken into account. The restrictions of the protected area led to many accusations and it was not approved" **[policy]**	2_MAN_L2
Access to knowledge and education	*na*	policy	1	"The new environmental laws were very shocking. Nobody knew why I suddenly couldn't do what I had done all my life. It was an authoritarian rule, far removed from the reality and way of life of the people" **[policy]**	3_NEI_B1
				"When our land was declared a protected area there was no information, people simply started to be fined for doing things, leading to a major conflict" **[policy]**	3_NEI_B2
Access to recreation and leisure	*na*	demography, policy	2	"Until five years ago we had our bathing places on the beach [...] but now on Saturdays and Sundays we don't go at all because our places are occupied and there are only problems" **[demography]**	10_COU_2
Enjoyment of natural beauty	land-use change	*na*	1	"Industrial agriculture extends to the mouths of dry rivers, where there are sedimentary deposits, altering the landscape and its values" **[land-use change]**	5_RES_2
Sense of cultural identity	land-use change	*na*	1	"People don't see the SPA [Special Protection Area] as a positive thing because they have seen how the traditional activities and heritage they enjoy being curtailed" **[land-use change]**	6_TOU_2

COU: neighbourhood council, **RES:** researcher, **NEI_B:** basin-born neighbour, **ORG_E:** environmental non-profit organisation, **ORG_S:** social non-profit organisation, **CRO_T:** traditional crop farmer, **CRO_I:** industrial crop farmer, **MAN_L:** land environmental manager, **NEI_B:** basin-born neighbour, **TOU:** tourism sector; ***na***: not applicable

Figure S1

Relationships between beneficial Nature's Contributions to People (NCP) and dimensions of a Good Quality of Life (GQL) as perceived by interviewees. The data are based on interviews with 37 representatives of the ten most influential social groups of three dry rivers in the Region of Murcia (Spain). Using the conceptual framework of the Intergovernmental Platform on Biodiversity and Ecosystem Services (IPBES), beneficial NCP (Díaz et al. 2018) and GQL dimensions (Brondízio et al. 2019) were coded. Relationships between them were identified using a co-occurrence function. This function produced a matrix in which each element represented the number of interviewees who reported a relationship between a beneficial NCP and a GQL dimension. For example, the relationship between food and feed (beneficial NCP) and livelihood and income security (GQL dimension) was reported by 14 interviewees. Relationships reported by many interviewees are depicted in red, by few interviewees in yellow, and by none in green. The category "unspecified NCP" refers to various NCP that were not specified by the interviewees but which have an impact on GQL dimensions. For example, the quote "this environment promotes social relations" does not specify which NCP promote social relations. Unspecified NCP were not considered in the main manuscript because of their ambiguity. Non-mat: non-material, Ot: other

			GQL dimensions													
			Material					Non-material								
			Food security	Water security	Shelter	Livelihood and income security	Health	Good social relationships	Equity	Sense of cultural identity	Personal and physical security	Freedom of participation	Freedom of choice and action	Access to knowledge and education	Access to recreation and leisure	Enjoyment of natural beauty
Beneficial NCP	Material	Energy	0	0	0	1	1	0	0	0	0	0	0	0	0	0
		Food and feed	18	4	1	14	2	5	1	3	2	3	5	1	1	0
		Materials, companionship and labour	0	1	0	5	0	5	0	2	0	1	0	0	0	0
		Medicinal and genetic resources	0	0	0	0	0	0	0	0	0	0	0	0	0	0
	Regulating	Habitat creation and maintenance	0	0	0	0	0	0	0	0	0	0	1	0	0	1
		Regulation of air quality	0	0	0	0	0	0	0	0	0	0	0	0	0	0
		Regulation of climate	0	0	2	0	1	0	0	0	0	0	0	0	0	0
		Regulation of freshwater quantity, location and timing	1	12	0	2	0	0	0	1	0	0	0	0	0	0
		Regulation of freshwater quality	0	1	0	0	1	0	0	0	0	0	0	0	0	0
		Formation and protection of soils	0	0	0	0	0	1	0	0	0	0	0	0	0	0
		Regulation of hazards and extreme events	0	0	0	0	0	0	0	0	2	0	0	0	0	0
		Regulation of detrimental organisms	0	0	0	0	0	0	0	0	0	0	0	0	0	0
	Non-mat.	Learning and inspiration	1	2	0	5	0	8	0	2	0	4	4	6	3	4
		Physical and psychological experiences	1	0	1	3	10	4	0	7	0	4	2	1	17	13
		Supporting identities	1	0	0	2	1	5	0	10	0	3	1	0	2	2
	Other	Connecting path	2	0	2	1	0	4	1	1	0	1	1	0	0	0
		Unspecified	0	0	1	2	1	18	3	0	1	15	3	0	0	0

Figure S2

Relationships between beneficial Nature's Contributions to People (NCP) and dimensions of a Good Quality of Life (GQL) as perceived by interviewees **in the past**. The data are based on interviews with 37 representatives of the ten most influential social groups of three dry rivers in the Region of Murcia (Spain). Using the conceptual framework of the Intergovernmental Platform on Biodiversity and Ecosystem Services (IPBES), beneficial NCP (Díaz et al. 2018) and GQL dimensions (Brondízio et al. 2019) were coded. Relationships between them were identified using a co-occurrence function. This function produced a matrix in which each element represented the number of interviewees who reported a relationship between a beneficial NCP and a GQL dimension in the past. For example, the relationship between food and feed (beneficial NCP) and livelihood and income security (GQL dimension) was reported by ten interviewees. Relationships reported by many interviewees are depicted in red, by few interviewees in yellow, and by none in green. Non-mat: non-material, Ot: other

			GQL dimensions													
			Material					Non-material								
			Food security	Water security	Shelter	Livelihood and income security	Health	Good social relationships	Equity	Sense of cultural identity	Personal and physical security	Freedom of participation	Freedom of choice and action	Access to knowledge and education	Access to recreation and leisure	Enjoyment of natural beauty
Beneficial NCP	Material	Energy	0	0	0	1	0	0	0	0	0	0	0	0	0	0
		Food and feed	14	4	1	10	0	2	1	3	0	2	2	1	0	0
		Materials, companionship and labour	0	1	0	5	0	5	0	2	0	0	0	0	0	0
		Medicinal and genetic resources	0	0	0	0	0	0	0	0	0	0	0	0	0	0
	Regulating	Habitat creation and maintenance	0	0	0	0	0	0	0	0	0	0	0	0	0	1
		Regulation of air quality	0	0	0	0	0	0	0	0	0	0	0	0	0	0
		Regulation of climate	0	0	1	0	0	0	0	0	0	0	0	0	0	0
		Regulation of freshwater quantity, location and timing	1	11	0	2	0	0	0	1	0	0	0	0	0	0
		Regulation of freshwater quality	0	1	0	0	1	0	0	0	0	0	0	0	0	0
		Formation and protection of soils	0	0	0	0	0	1	0	0	0	0	0	0	0	0
		Regulation of hazards and extreme events	0	0	0	0	0	0	0	0	2	0	0	0	0	0
		Regulation of detrimental organisms	0	0	0	0	0	0	0	0	0	0	0	0	0	0
	Non-mat.	Learning and inspiration	0	2	0	4	0	5	0	1	0	0	1	1	1	0
		Physical and psychological experiences	0	0	0	0	0	0	0	2	0	1	1	0	2	1
		Supporting identities	0	0	0	2	0	2	0	2	0	0	0	0	0	0
	Ot.	Connecting path	2	0	2	1	0	4	1	0	0	0	0	0	0	0

Figure S3

Relationships between beneficial Nature's Contributions to People (NCP) and dimensions of a Good Quality of Life (GQL) as perceived by interviewees **in the present**. The data are based on interviews with 37 representatives of the ten most influential social groups of three dry rivers in the Region of Murcia (Spain). Using the conceptual framework of the Intergovernmental Platform on Biodiversity and Ecosystem Services (IPBES), beneficial NCP (Díaz et al. 2018) and GQL dimensions (Brondízio et al. 2019) were coded. Relationships between them were identified using a co-occurrence function. This function produced a matrix in which each element represented the number of interviewees who reported a relationship between a beneficial NCP and a GQL dimension in the present. For example, the relationship between food and feed (beneficial NCP) and livelihood and income security (GQL dimension) was reported by eight interviewees. Relationships reported by many interviewees are depicted in red, by few interviewees in yellow, and by none in green. Non-mat: non-material, Ot: other

		GQL dimensions													
		Material					Non-material								
		Food security	Water security	Shelter	Livelihood and income security	Health	Good social relationships	Equity	Sense of cultural identity	Personal and physical security	Freedom of participation	Freedom of choice and action	Access to knowledge and education	Access to recreation and leisure	Enjoyment of natural beauty
Beneficial NCP – Material	Energy	0	0	0	0	1	0	0	0	0	0	0	0	0	0
	Food and feed	8	0	0	8	2	3	0	1	2	1	3	0	1	0
	Materials, companionship and labour	0	0	0	0	0	0	0	0	0	1	0	0	0	0
	Medicinal and genetic resources	0	0	0	0	0	0	0	0	0	0	0	0	0	0
Regulating	Habitat creation and maintenance	0	0	0	0	0	0	0	0	0	0	1	0	0	1
	Regulation of air quality	0	0	0	0	0	0	0	0	0	0	0	0	0	0
	Regulation of climate	0	0	1	0	1	0	0	0	0	0	0	0	0	0
	Regulation of freshwater quantity, location and timing	0	2	0	0	0	0	0	0	0	0	0	0	0	0
	Regulation of freshwater quality	0	0	0	0	0	0	0	0	0	0	0	0	0	0
	Formation and protection of soils	0	0	0	0	0	0	0	0	0	0	0	0	0	0
	Regulation of hazards and extreme events	0	0	0	0	0	0	0	0	2	0	0	0	0	0
	Regulation of detrimental organisms	0	0	0	0	0	0	0	0	0	0	0	0	0	0
Non-mat.	Learning and inspiration	1	1	0	1	0	3	0	1	0	4	3	5	2	4
	Physical and psychological experiences	1	0	1	3	10	4	0	5	0	4	1	1	15	13
	Supporting identities	1	0	0	1	1	5	0	8	0	3	1	0	2	2
Ot	Connecting path	0	0	0	0	0	1	0	1	0	1	1	0	0	0

Figure S4

Relationships between detrimental Nature's Contributions to People (NCP) and dimensions of a Good Quality of Life (GQL) as perceived by interviewees. The data are based on interviews with 37 representatives of the ten most influential social groups of three dry rivers in the Region of Murcia (Spain). Using the conceptual framework of the Intergovernmental Platform on Biodiversity and Ecosystem Services (IPBES), detrimental NCP (Díaz et al. 2018) and GQL dimensions (Brondízio et al. 2019) were coded. Relationships between them were identified using a co-occurrence function. This function produced a matrix in which each element represented the number of interviewees who reported a relationship between a detrimental NCP and a GQL dimension. For example, the relationship between regulation of hazards and extreme events (detrimental NCP) and personal and physical security (GQL dimension) was reported by eleven interviewees. Relationships reported by many interviewees are depicted in red, by few interviewees in yellow, and by none in green. Non-mat: non-material, Ot: other

		GQL dimensions													
		Material					Non-material								
Detrimental NCP		Food security	Water security	Shelter	Livelihood and income security	Health	Good social relationships	Equity	Sense of cultural identity	Personal and physical security	Freedom of participation	Freedom of choice and action	Access to knowledge and education	Access to recreation and leisure	Enjoyment of natural beauty
Material	Energy	0	0	0	0	0	0	0	0	0	0	0	0	0	0
	Food and feed	0	0	0	0	0	0	0	0	0	0	0	0	0	0
	Materials, companionship and labour	0	0	0	0	0	0	0	0	0	0	0	0	0	0
	Medicinal and genetic resources	0	0	0	0	0	0	0	0	0	0	0	0	0	0
Regulating	Habitat creation and maintenance	0	0	0	0	0	0	0	0	0	0	0	0	0	0
	Regulation of air quality	0	0	0	0	0	0	0	0	0	0	0	0	0	0
	Regulation of climate	0	0	0	0	0	0	0	0	0	0	0	0	0	0
	Regulation of freshwater quantity, location and timing	0	0	0	0	0	0	0	0	9	0	0	0	0	0
	Regulation of freshwater quality	0	0	0	0	0	0	0	0	0	0	0	0	0	0
	Formation and protection of soils	0	0	0	0	0	0	0	0	0	0	0	0	0	0
	Regulation of hazards and extreme events	0	0	0	1	0	0	0	0	11	0	0	0	1	0
	Regulation of detrimental organisms	0	0	0	1	2	0	0	0	7	0	0	0	0	0
Non-mat.	Learning and inspiration	0	0	0	0	0	0	0	0	0	0	0	0	0	0
	Physical and psychological experiences	0	0	0	0	0	0	0	0	0	0	0	0	3	1
	Supporting identities	0	0	0	0	0	0	0	0	0	0	0	0	0	0
Ot.	Connecting path	0	0	0	0	0	0	0	0	0	0	0	0	0	0

Figure S5

Relationships between detrimental Nature's Contributions to People (NCP) and dimensions of a Good Quality of Life (GQL) as perceived by interviewees **in the past**. The data are based on interviews with 37 representatives of the ten most influential social groups of three dry rivers in the Region of Murcia (Spain). Using the conceptual framework of the Intergovernmental Platform on Biodiversity and Ecosystem Services (IPBES), detrimental NCP (Díaz et al. 2018) and GQL dimensions (Brondízio et al. 2019) were coded. Relationships between them were identified using a co-occurrence function. This function produced a matrix in which each element represented the number of interviewees who reported a relationship between a detrimental NCP and a GQL dimension in the past. For example, the relationship between the regulation of hazards and extreme events (detrimental NCP) and personal and physical security (GQL dimension) was reported by four interviewees. Relationships reported by many interviewees are depicted in red, by few interviewees in yellow, and by none in green. Non-mat: non-material, Ot: other

			GQL dimensions													
			Material					Non-material								
			Food security	Water security	Shelter	Livelihood and income security	Health	Good social relationships	Equity	Sense of cultural identity	Personal and physical security	Freedom of participation	Freedom of choice and action	Access to knowledge and education	Access to recreation and leisure	Enjoyment of natural beauty
Detrimental NCP	Material	Energy	0	0	0	0	0	0	0	0	0	0	0	0	0	0
		Food and feed	0	0	0	0	0	0	0	0	0	0	0	0	0	0
		Materials, companionship and labour	0	0	0	0	0	0	0	0	0	0	0	0	0	0
		Medicinal and genetic resources	0	0	0	0	0	0	0	0	0	0	0	0	0	0
	Regulating	Habitat creation and maintenance	0	0	0	0	0	0	0	0	0	0	0	0	0	0
		Regulation of air quality	0	0	0	0	0	0	0	0	0	0	0	0	0	0
		Regulation of climate	0	0	0	0	0	0	0	0	0	0	0	0	0	0
		Regulation of freshwater quantity, location and timing	0	0	0	0	0	0	0	0	4	0	0	0	0	0
		Regulation of freshwater quality	0	0	0	0	0	0	0	0	0	0	0	0	0	0
		Formation and protection of soils	0	0	0	0	0	0	0	0	0	0	0	0	0	0
		Regulation of hazards and extreme events	0	0	0	1	0	0	0	0	4	0	0	0	0	0
		Regulation of detrimental organisms	0	0	0	0	1	0	0	0	0	0	0	0	0	0
	Non-mat.	Learning and inspiration	0	0	0	0	0	0	0	0	0	0	0	0	0	0
		Physical and psychological experiences	0	0	0	0	0	0	0	0	0	0	0	0	0	0
		Supporting identities	0	0	0	0	0	0	0	0	0	0	0	0	0	0
	Ot.	Connecting path	0	0	0	0	0	0	0	0	0	0	0	0	0	0

Figure S6

Relationships between detrimental Nature's Contributions to People (NCP) and dimensions of a Good Quality of Life (GQL) as perceived by interviewees **in the present**. The data are based on interviews with 37 representatives of the ten most influential social groups of three dry rivers in the Region of Murcia (Spain). Using the conceptual framework of the Intergovernmental Platform on Biodiversity and Ecosystem Services (IPBES), detrimental NCP (Díaz et al. 2018) and GQL dimensions (Brondízio et al. 2019) were coded. Relationships between them were identified using a co-occurrence function. This function produced a matrix in which each element represented the number of interviewees who reported a relationship between a detrimental NCP and a GQL dimension in the present. For example, the relationship between the regulation of hazards and extreme events (detrimental NCP) and personal and physical security (GQL dimension) was reported by nine interviewees. Relationships reported by many interviewees are depicted in red, by few interviewees in yellow, and by none in green. Non-mat: non-material, Ot: other

			GQL dimensions													
			Material					Non-material								
			Food security	Water security	Shelter	Livelihood and income security	Health	Good social relationships	Equity	Sense of cultural identity	Personal and physical security	Frredom of participation	Freedom of choice and action	Access to knowledge and education	Access to recreation and leisure	Enjoyment of natural beauty
Detrimental NCP	Material	Energy	0	0	0	0	0	0	0	0	0	0	0	0	0	0
		Food and feed	0	0	0	0	0	0	0	0	0	0	0	0	0	0
		Materials, companionship and labour	0	0	0	0	0	0	0	0	0	0	0	0	0	0
		Medicinal and genetic resources	0	0	0	0	0	0	0	0	0	0	0	0	0	0
	Regulating	Habitat creation and maintenance	0	0	0	0	0	0	0	0	0	0	0	0	0	0
		Regulation of air quality	0	0	0	0	0	0	0	0	0	0	0	0	0	0
		Regulation of climate	0	0	0	0	0	0	0	0	0	0	0	0	0	0
		Regulation of freshwater quantity, location and timing	0	0	0	0	0	0	0	0	8	0	0	0	0	0
		Regulation of freshwater quality	0	0	0	0	0	0	0	0	0	0	0	0	0	0
		Formation and protection of soils	0	0	0	0	0	0	0	0	0	0	0	0	0	0
		Regulation of hazards and extreme events	0	0	0	0	0	0	0	0	9	0	0	0	1	0
		Regulation of detrimental organisms	0	0	0	1	1	0	0	0	7	0	0	0	0	0
	Non-mat.	Learning and inspiration	0	0	0	0	0	0	0	0	0	0	0	0	0	0
		Physical and phychological experiences	0	0	0	0	0	0	0	0	0	0	0	0	3	1
		Supporting identities	0	0	0	0	0	0	0	0	0	0	0	0	0	0
	Ot.	Connecting path	0	0	0	0	0	0	0	0	0	0	0	0	0	0

Figure S7

Relationships between drivers of change and dimensions of a Good Quality of Life (GQL) as perceived by interviewees. The data are based on interviews with 37 representatives of the ten most influential social groups of three dry rivers in the Region of Murcia (Spain). Using the conceptual framework of the Intergovernmental Platform on Biodiversity and Ecosystem Services (IPBES), drivers (Elbakidze et al. 2018) and GQL dimensions (Brondízio et al. 2019) were coded. Relationships between them were identified using a co-occurrence function. This function produced a matrix in which each element represented the number of interviewees who reported a relationship between a driver type and a GQL dimension. For example, the relationship between policy (driver) and freedom of choice and action (GQL dimension) was reported by 15 interviewees. Relationships reported by many interviewees are depicted in red, by few interviewees in yellow and by none in green.

			GQL dimensions													
			Material					Non-material								
			Food security	Water security	Shelter	Livelihood and income security	Health	Good social relationships	Equity	Sense of cultural identity	Personal and physical security	Freedom of participation	Freedom of choice and action	Access to knowledge and education	Access to recreation and leisure	Enjoyment of natural beauty
Drivers	Direct	Natural resources extraction	0	1	0	0	0	0	0	0	0	0	1	0	0	0
		Invasive alien species	0	0	0	0	0	0	0	0	3	0	0	0	0	0
		Pollution	0	0	0	1	2	7	1	0	3	0	3	0	0	0
		Land-use change	0	0	0	5	1	4	0	1	3	3	5	0	0	1
		Climate change	0	1	0	0	0	0	0	0	0	0	0	0	0	0
	Indirect	Demography	0	0	0	10	0	3	0	0	2	0	4	0	1	0
		Economy	0	0	0	9	0	2	1	0	0	0	1	0	0	0
		Policy	0	0	0	2	0	4	2	0	2	3	15	2	1	0
		Science and technology	0	0	0	0	1	0	0	0	0	0	0	0	0	0
		Culture and religion	0	0	0	5	0	2	0	0	0	1	1	0	0	0

References

Brondízio, E., S. Diaz, Josef Settele, Hien T. Ngo, M. Gueze, Y. Aumeeruddy-Thomas, X. Bai, A. Geschke, et al. 2019. Chapter 1 Assessing a planet in transformation: Rationale and approach of the IPBES Global Assessment on Biodiversity and Ecosystem Services. In *The global assessment report on biodiversity and ecosystem services*, ed. E. S. Brondízio, J Settele, S. Díaz, and H.T. Ngo, 1–48. Bonn, Germany: IPBES secretariat. doi:10.5281/ZENODO.3831852.

Díaz, S., U. Pascual, M. Stenseke, B. Martín-López, R. T. Watson, Z. Molnár, R. Hill, K. M. A. Chan, et al. 2018. Assessing nature's contributions to people. *Science* 359: 270–272. doi:10.1126/science.aap8826.

Elbakidze, M., T. Hahn, and N. E. Zimmermann. 2018. Chapter 4. Direct and indirect drivers of change in biodiversity and nature's contributions to people. In *The IPBES regional assessment report on biodiversity and ecosystem services for Europe and Central Asia*, ed. M. Rounsevell, M. Fischer, A. Torre-Marín Rando, and A. Mader, 385–570. Bonn (Germany): IPBES secretariat.

Nicolás-Ruiz, N., M. L. Suárez Alonso, and M. R. Vidal-Abarca. 2021. Contributions of dry rivers to human well-being: A global review for future research. *Ecosystem Services* 50: 101307. doi:10.1016/j.ecoser.2021.101307.

Nicolás-Ruiz, N., C. Quintas-Soriano, M. L. Suárez, and M. R. Vidal-Abarca. 2023. Co-production of nature's contributions to people in dry rivers: a case study in Murcia, Spain. *Ecosystems and People* 19. doi:https://doi.org/10.1080/26395916.2023.2288953.

Vidal-Abarca, M., R. Gómez, M. Sánchez-Montoya, M. Arce, N. Nicolás, and M. Suárez. 2020. Defining Dry Rivers as the Most Extreme Type of Non-Perennial Fluvial Ecosystems. *Sustainability* 12: 7202. doi:10.3390/su12177202.

Vidal-Abarca, M. R., N. Nicolás-Ruiz, M. del M. Sánchez-Montoya, and M. L. Suárez Alonso. 2022. Ecosystem services provided by dry river socio-ecological systems and their drivers of change. *Hydrobiologia* 850: 2585–2607. doi:10.1007/s10750-022-04915-8.
